# Natural Yeast Promoter Variants Reveal Epistasis in the Generation of Transcriptional-Mediated Noise and Its Potential Benefit in Stressful Conditions

**DOI:** 10.1093/gbe/evv047

**Published:** 2015-03-11

**Authors:** Jian Liu, Hélène Martin-Yken, Frédéric Bigey, Sylvie Dequin, Jean-Marie François, Jean-Pascal Capp

**Affiliations:** ^1^Laboratoire d’Ingénierie des Systèmes Biologiques et des Procédés, UMR CNRS 5504, UMR INRA 792, INSA/Université de Toulouse, France; ^2^INRA, UMR 1083 Sciences Pour l’Œnologie, Montpellier, France

**Keywords:** stochastic gene expression, phenotypic variability and heterogeneity, stress resistance and tolerance, industrial wine yeast, copper

## Abstract

The increase in phenotypic variability through gene expression noise is proposed to be an evolutionary strategy in selective environments. Differences in promoter-mediated noise between *Saccharomyces cerevisiae* strains could have been selected for thanks to the benefit conferred by gene expression heterogeneity in the stressful conditions, for instance, those experienced by industrial strains. Here, we used a genome-wide approach to identify promoters conferring high noise levels in the industrial wine strain EC1118. Many promoters of genes related to environmental factors were identified, some of them containing genetic variations compared with their counterpart in the laboratory strain S288c. Each variant of eight promoters has been fused to yeast-Enhanced Green Fluorescent Protein and integrated in the genome of both strains. Some industrial variants conferred higher expression associated, as expected, with lower noise, but other variants either increased or decreased expression without modifying variability, so that they might exhibit different levels of transcriptional-mediated noise at equal mean. At different induction conditions giving similar expression for both variants of the *CUP1* promoter, we indeed observed higher noise with the industrial variant. Nevertheless, this difference was only observed in the industrial strain, revealing epistasis in the generation of promoter-mediated noise. Moreover, the increased expression variability conferred by this natural yeast promoter variant provided a clear benefit in the face of an environmental stress. Thus, modulation of gene expression noise by a combination of promoter modifications and *trans*-influences might be a possible adaptation mechanism in yeast.

## Introduction

Large fluctuations in gene expression levels among individual cells exist in isogenic populations even under constant environmental conditions (Elowitz et al. 2002; Blake et al. 2003; Raser and O’Shea 2004). This gene expression variability, also called noise, is due to stochastic fluctuations at the molecular level and is now recognized as widely involved in major biological phenomena because it can have profound phenotypic consequences (Raj and van Oudenaarden 2008; Balazsi et al. 2011). Stochastic gene expression could be especially advantageous in that it would allow heterogeneous phenotypes to appear among genetically identical cells, enabling a population to contain subpopulations with different behaviors. Thus, it would favor proliferation of preadapted cells through noise when a stress appears or when the environment fluctuates (Blake et al. 2006; Smith et al. 2007; Acar et al. 2008; Fraser and Kaern 2009; Ito et al. 2009; Lidstrom and Konopka 2010; Ackermann 2013).

As noise could be advantageous in regard to challenging environments, are genes related to stress responses or other environmental factors noisier than housekeeping genes? In 2006, two studies showed that protein-specific differences in noise exist and are strongly correlated with modes of transcription and protein functions (Bar-Even et al. 2006; Newman et al. 2006). Proteins responding to environmental changes are “noisy” whereas those involved in housekeeping processes are not. Noise levels seem to have been selected for depending on the costs and potential benefits of this variation. Noise is also minimized for essential and complex-forming proteins (Fraser et al. 2004; Lehner 2008) whereas TATA box-containing genes, mainly associated with stress or other environmental factors, are noisier (Newman et al. 2006). These data are in accordance with works showing how selection influences phenotypic fluctuations in evolutionary experiments. A study on *Escherichia coli* provides evidence that mutants with similar mean and a larger degree of phenotypic variability due to variations in mRNA abundance emerged under strong selection pressure, together with mutants harboring increased mean and no increased variability, even if the environment is not fluctuating (Ito et al. 2009). Here, the increase in phenotypic heterogeneity that probably occurred through transcriptional-mediated noise in gene expression was a relevant evolutionary strategy because the larger distribution of the more variable mutants could result in a similar survival probability than for mutants with increased mean and narrow distribution. Such variability in expression confers a benefit in constant stressful conditions for yeast populations because it generates, in the absence of stress, a phenotypic diversity that makes the presence of preadapted cells more probable (Blake et al. 2006; Smith et al. 2007). Moreover, it appears to also be the case in fluctuating environments (Acar et al. 2008). Nevertheless few studies have investigated fitness effects of noise in gene expression (Viney and Reece 2013), especially in eukaryotes, and it was only with artificially manipulated promoters conferring different levels of noise in the expression of proteins involved in stress response or antibiotics resistance (Blake et al. 2006; Smith et al. 2007). Whether natural promoters have naturally evolved toward different levels of noise because of the potential benefits conferred by noise-mediated phenotypic variability is still unknown (Ackermann 2013; Viney and Reece 2013; Holland et al. 2014).

Three main promoter elements can affect noise in gene expression at the transcriptional level in eukaryotes: Nucleosome binding sites, TATA boxes, and transcription factors binding sites (Sanchez et al. 2013). By randomly mutating or rationally modifying these sequences, several studies have already produced promoter variants that harbor different noise levels, sometimes at similar mean expression levels (Murphy et al. 2010; Hornung et al. 2012; Carey et al. 2013; Dadiani et al. 2013; Sanchez and Golding 2013; Sharon et al. 2014). Nevertheless, no particular study has tried to identify molecular adaptation of eukaryotic species through modulation of promoter-mediated variability. How natural genetic variation influences the level of noise in the expression of a single gene has already been shown in *Saccharomyces cerevisiae* and reproducible differences in noise were observed between divergent genetic backgrounds (Ansel et al. 2008). In particular, it was found that noise was highly heritable and placed under a complex genetic control mechanism. But the experimental strategy of this work led to identify differences in *trans*-acting factors on promoters, and not promoter sequence variations modifying gene expression variability.

Industrial *S. cerevisiae* strains provide a good model to study molecular adaptation to challenging environments. They have been selected for rapid fermentations and are specifically adapted to the stressful conditions of fermentation, characterized by high sugar content, high alcohol content, low pH, the presence of sulfites, limiting amounts of nitrogen, lipids and vitamins, anaerobiosis, and other environmental stresses. Although they are genetically highly related to their laboratory counterpart, the genetic basis of their technological properties as compared with laboratory yeast strains that are inefficient under these fermentation conditions is still largely unknown. Genome-wide approaches have received a strong interest in recent years to address the question of the adaptation of industrial wine yeasts to these specific conditions (Dunn et al. 2005, 2012; Novo et al. 2009; Ambroset et al. 2011; Dequin and Casaregola 2011; Salinas et al. 2012). Various mechanisms have been proposed to play a role in the adaptive evolution of wine yeasts, such as polyploidy, aneuploidy, chromosomal translocations, copy number variations, or horizontal gene transfer. It is also expected that sequence polymorphisms (single nucleotide polymorphisms [SNPs]) and insertions/deletions (INDELs) have a strong contribution to the observed properties.

Quantitative genetic analysis by quantitative trait loci (QTL) and expression QTL methods is mostly employed to identify the genetic determinants of phenotypic divergence among *S. cerevisiae* strains (Ambroset et al. 2011; Liti and Louis 2012; Brion et al. 2013; Fay 2013). Their evolution toward quantitative trait genes and nucleotides methods thanks to high throughput next-generation sequencing now provides powerful tools to study quantitative trait variations (Fay 2013). These technologies enable comparative genomics to characterize in-depth genetic variations between wine yeast strains (Borneman et al. 2013). Nevertheless, only mean expression values are considered in these methods. They cannot detect the influence of gene expression variability at the single cell level. A recent study defined a probabilistic trait locus (PTL) as “a locus that changes the probability that an individual expresses a given trait value in a given genomic and environmental context” and expression PTL as a locus affecting the expression variability of a given gene (Fehrmann et al. 2013). With these conceptual tools, the authors identified among different *S. cerevisiae* strains several loci modifying cell–cell variability in the expression of GFP fused to the *MET17* promoter (Fehrmann et al. 2013). In particular, genetic variants of yeast environmental sensors (plasma transporters) generate different noise levels in the expression of this downstream promoter, possibly through variability in pathway activation. These variants were found to either reduce or increase GFP expression variability in *trans*. However, the method does not allow identifying promoter modifications modifying noise in *cis*. Therefore this study identified for the first time natural genetic variants influencing noise in transcription in yeast, but only through effects on downstream genes in a cellular pathway. Moreover, as with most studies on gene expression variability (Viney and Reece 2013), it did not consider phenotypic consequences of noise modifications. Phenotypic consequences mediated by natural yeast genetic variants modifying gene expression variability have never been investigated.

As genes related to stress responses and external stimuli are associated with higher levels of cell–cell variability (Newman et al. 2006), and promoter-mediated noise seems to be evolvable and heritable (Ito et al. 2009), industrial *S. cerevisiae* strains might have evolved through promoter modifications toward higher transcriptional-mediated noise levels in the expression of genes involved in their technological traits and their survival in stressful environments. Here we screened for promoters conferring high expression variability in the sequenced industrial wine *S. cerevisiae* strain EC1118, in order to compare their sequence with the ones of their counterpart of the laboratory strain S288c. Our aim was to determine whether the observed genetic differences generate noise differences between the variants. By expressing yEGFP under the dependence of either the laboratory or the industrial variant of eight promoters in both strains, we notably showed that the industrial promoter variant of the yeast *CUP1* gene encoding metallothionein exhibits higher expression variability than the laboratory variant at equal mean expression level. We furthermore observed this difference only in the industrial strain, revealing epistasis in the generation of transcriptional-mediated noise. The combined influences of variations in *cis*- (promoter sequence) and *trans*-elements act to increase noise. Moreover, this enhanced promoter-mediated variability with the industrial variant improved survival of the population under constant selective conditions. Therefore, this promoter has probably naturally evolved toward higher noise because it increases the global fitness of the population in challenging environments. This study identified a possible adaptation mechanism in yeast by showing that natural promoter variants from different strains can confer different survival abilities in selective environments due to differences in the expression variability of the associated gene.

## Materials and Methods

### Yeast and Bacteria Strains and Growth Conditions

NEB 5-alpha competent *E. coli* (high efficiency) (New England Biolabs) was grown at 37 °C in LB medium containing 100 μg/ml ampicillin (Euromedex). Yeast strains were grown in YPD or YNB medium at 30 °C. CEN-PK (*MATa ura3-52*) was used to construct the genomic library (see below). Isolation of the commercial wine strain EC1118 haploid derivative 59A was described previously (Ambroset et al. 2011). 59A *MAT*α *Δamn1-loxP* (provided by V. Galeote, INRA SPO) and the S288c auxotrophic derivative BY4720 (*MATα lys2Δ0 trp1Δ63 ura3Δ0*) were used for mean expression and noise measurements at the genomic level. Both strains have been transformed by pJRL2 plasmids to integrate promoter variants fused to yEGFP in their *LEU2* locus (see below and supplementary table S6, Supplementary Material online, for the list of strains). Cells transformed with pJRL2 plasmids were grown on plates containing 200 µg/ml G418 (Euromedex). Induction by CuSO_4_ was performed either in YPD or in YNB medium.

### Construction of the Promoterless yEGFP-Coding Vectors

Three different promoterless yEGFP-coding vectors were constructed using the yEGFP-coding pUG35 centromeric plasmid as a backbone vector. The *MET25* promoter and the multiple cloning site upstream yEGFP of this vector were replaced by the *kanMX4* gene and a unique *Sna*BI restriction site (generating blunt ends). The *kanMX4* gene was polymerase chain reaction (PCR) amplified from the pfa6 vector to recombine within pUG35 (see primers in supplementary table S7, Supplementary Material online). The forward primer contained homology to the beginning of the *MET25* promoter of pUG35. The reverse primers contained homology to the end of the multiple cloning site of pUG35. Three different reverse primers were used to construct three different plasmids containing 0, 1, or 2 additional base(s) between *Sna*BI and the start codon of yEGFP. The resulting plasmids were promoterless yEGFP-coding vectors containing a *Sna*BI restriction site before the start codon of yEGFP to fuse genomic fragments to yEGFP.

### Construction of the yEGFP-Fused Genomic DNA Library

The 59A genomic DNA was isolated using the MasterPure Yeast DNA Purification Kit (Epicentre) and treated with RNAse A. The obtained DNA was fragmented independently by the two 4-cutter restriction enzymes *Rsa*I and *Alu*I generating blunt ends compatible with the ends generated by *Sna*BI in the promoterless yEGFP-coding vectors. Reaction times and enzyme concentrations were optimized to produce DNA fragments ranging from 500 bp to 3 kb. For *Rsa*I, 1.5 µg DNA was digested during 15 min by 1 U enzyme. For *Alu*I, 3 µg DNA has been digested during 30 min by 0.5 U enzyme.

Fragments from 500 bp to 3 kb generated by each enzyme were gel extracted (QIAquick Gel Extraction Kit, Qiagen) and ligated independently by the DNA ligase T4 (overnight, 16 °C, Quick Ligation Kit, New England Biolabs) with each of the three promoterless yEGFP-coding vectors previously digested by *Sna*BI, dephosphorylated (Antarctic Phosphatase, New England Biolabs), and purified. The ratio (vector:inserts) used for the six ligation reactions (two enzymes, three vectors) was 1:2,6 because it yielded a higher number of transformants. The number of transformants required for each (enzyme:vector) pair to give a 99% confidence level that all sequences of the genome are represented with a mean insert size of 2 kb was 30,000. This number was multiplied by 2 because any fragment can be inserted in both senses. Thus 60,000 transformants for each (enzyme:vector) pair were independently obtained after ligation, transformation of competent *E. coli* cells using standard methodology, and growth on selective LB medium. Then, cells were harvested and pooled to isolate the plasmids from the six bulk cultures (GenElute HP Plasmid Midiprep Kit, Sigma-Aldrich). Redigestion with *SnaBI* was performed to linearize empty promoterless yEGFP-coding plasmids. Plasmids from each (enzyme:vector) pair were then retransformed in the laboratory yeast strain CEN-PK using classical lithium acetate method. Again 60,000 transformants for each (enzyme:vector) pair were independently obtained after growth in selective medium (YNB ura-). Finally, the transformants originated from the six (enzyme:vector) pairs were pooled together at similar Optical Density (OD) and equal volume to form the final library used for fluctuating selection.

### Fluctuating Selection Using Cell Sorting

The method described by Freed et al. (2008) has been adapted to *S. cerevisiae*. An overnight culture of the population containing the genomic library was diluted to OD = 0.5 and cells were grown for around 5 h to reach exponential growth (OD = 2) (YNB ura- medium). Cultures were spun down at 3,000 g for 5 min at 4 °C. Growth media were removed and cultures were resuspended in ice-cold Phosphate Saline Buffer (PBS). Cells were then kept on ice until cell sorting. The yEGFP-fused genomic library was subjected to fluctuating selection on fluorescence intensity, where selection for bright cells alternated with selection for dim cells using fluorescence-activated cell sorting (FACS) performed by the FACS Calibur associated with the Cellquest sorting software (Becton Dickinson). On the first day, a gate was drawn to include only the fluorescent cells (around 4%). 1 × 10^5^ cells were collected into a sterile Falcon tube. Cells were collected at medium flow rate and sorted on the basis of “single cell” and “purity.” After sorting, cells were spun at 3,000 g for 10 min and any FACS buffer was removed. Cells were resuspended in 1-ml YNB ura- medium and grown overnight. The following day the process was repeated but the gate included only the lowest 5% of cells expressing yEGFP. This process was repeated for a total of seven rounds of selection, with gates being drawn for selected populations in a fluctuating manner with alternatively the highest or the lowest 5% of yEGFP expression in the gate. After the fourth round of selection, cells were placed at 4 °C for 48 h. After this time, selection was resumed as normal until the seventh round. After all rounds of selection were completed, the populations were plated on YNB ura- agar plates, and single colonies were randomly selected to confirm the enrichment in clones with high noise in yEGFP expression (see below). Because not all the clones harbored noisy yEGFP expression, a screening was needed to sequence plasmids from the noisiest clones only. Single clones were randomly selected, grown in 96-well plates overnight in YNB ura- medium and prepared and analyzed as described below. Two individual subclones were also reisolated from each selected clone to analyze whether the noise conferred by the genomic fragment was a stable property of the plasmid.

### Flow Cytometry Analysis

For each clone from the selected library, 10^5^ cells were analyzed for yEGFP expression on FACS Calibur (Becton Dickinson). Analysis of cytometry data was performed by the Cellquest software (Becton Dickinson). Calculation of variation in yEGFP expression was performed as followed to limit the influence of cellular aggregates, cell detritus, and undefined values: For each clone, a gate was created on the forward scatter (FSC) and side scatter (SSC) dot plot to exclude extreme or zero values from total counts and to include only a population of cells homogeneous in terms of size, shape, and cellular complexity. A single gate size was chosen for all analyses in order to maintain a conservative estimate of noise. The coefficient of variation (CV) was calculated for fluorescence in this gate. For some clones, a smaller gate was also applied on the densest subset of cells using the FSC/SSC density plot. This gating lowered average CV values because it minimizes “extrinsic” noise due to physiological differences between cells. This allowed verifying that CV was mainly due to “intrinsic” noise due to stochastic phenomena at the molecular level.

Analysis of mean and noise levels at the genomic level after integration of promoter variants fused to yEGFP in the *LEU2* locus of BY4720 and 59A was performed on the Attune Acoustic Flow Cytomoter (Life Technologies). For each strain, 10^5^ cells were analyzed for yEGFP expression. Analysis of cytometry data was performed by the Attune software (Life Technologies). A gate containing at least 10^4^ cells for robust analysis was applied on the densest subset of cells using the FSC/SSC density plot. The same gate was used to measure mean and noise levels conferred by the variants of a given promoter in a given strain in order to maintain a conservative estimate of noise.

### Genomic Integration of the yEGFP-Fused Promoter Variants

The BY4720 or 59A genomic DNA was isolated using the MasterPure Yeast DNA Purification Kit (Epicentre). The primers listed in supplementary table S7, Supplementary Material online, were used to amplify the *BMH1, BMH2, GNP1, YCK2, CAN1, HAC1, AGP2,* and *CUP1* promoters from both strains (1,000 bp upstream of the start codon except for *BMH2*: 1,742 bp). We inserted each promoter independently in a yeast chromosomal integration vector pJRL2 (Addgene) replacing the *LEU2* chromosomal locus by homologous recombination (Raser and O’Shea 2004). We modified the selection cassette in the pJRL2 vector: The *his–URA3–kanR–his* sequence was replaced by *kanMX4* only isolated from the pfa6 vector (using *Bgl*II and *Kpn*I). The *YFP* gene where a Kozak sequence had been introduced (Raser and O’Shea 2004) was replaced by yEGFP without Kozak sequence after yEGFP PCR amplification from the pUG35 vector and insertion within pJRL2 using *Eco*RI and *Not*I.

Thus yEGFP was fused to the *PHO5* promoter localized between the *Sal*I and *Eco*RI restriction sites in pJRL2. We then replaced the *PHO5* promoter by each promoter variant using forward primers containing a *Sal*I restriction site and reverse primers containing an *Eco*RI restriction site. The resulting 16 plasmids (two variants of eight promoters) were linearized by *Asc*I digestion (restriction site between the regions homologous to the *LEU2* locus) and transformed in both strains using the lithium acetate method. Recombinants were selected on YPD + G418 agar plates and insertion was verified. The 32 strains were submitted to the same experimental procedure to measure mean expression and noise levels in optimal growth conditions and exponential phase: An overnight culture was diluted to OD = 0.5 in the morning and cells were grown for around 5 h to reach exponential growth (OD = 2) (YPD + G418 medium). Cultures were spun down at 10,000 g for 30 s at 4 °C. Growth media were removed and cultures were resuspended in ice-cold PBS. Cells were then kept on ice until analysis.

### Directed Modification of the *pCUP1_S__288__c_* Variant

Directed mutagenesis with PCR amplification, *Dpn*I digestion, and transformation were classically performed to insert point mutations in the *pCUP1_S__288__c_* variant. Primers used are listed in supplementary table S7, Supplementary Material online. For the deletion, the pJRL2 plasmids containing *pCUP1_S__288__c_*-yEGFP and *pCUP1_EC__1118_*-yEGFP were digested with *Xba*I (cutting between SNP3 and the deletion) and *Sac*I (cutting downstream yEGFP) and the deletion was introduced into *pCUP1_S__288__c_* by exchanging the fragments.

### Induction of the *CUP1* Promoter Variants by Copper

An overnight culture was diluted to OD = 0.3 and cells were grown in YPD or YNB medium until exponential phase (4 h) before adding CuSO_4_ (ProLabo). Time-dependent induction was measured in 20 µM CuSO_4_ during up to 3 h and concentration-dependent induction was measured after 1 h in concentrations up to 50 µM CUSO_4_. Strains were then analyzed by flow cytometry as described above. When induction conditions giving similar mean expression levels for the different *CUP1* promoter variants were determined, experiments were reproduced at fixed amounts of time and concentration for a given variant. Overnight induction was measured after dilution of a copper-induced overnight culture to OD = 0.3 and growth in the same CuSO_4_ concentration during 5 h.

### Growth in Phleomycin-Containing Medium

The *ZeoR* gene was amplified with primers containing *Eco*RI (forward) and *Not*I (reverse) restriction sites. Then, yEGFP was removed by *Eco*RI/*Not*I digestion from the integration plasmids containing *pCUP1_S__288__c_*-yEGFP or *pCUP1_EC__1118_*-yEGFP to be replaced by *ZeoR*. Plasmids were then integrated in the *LEU2* locus in 59A as described above. Individual colonies of *ZeoR*-expressing strains were used to inoculate YNB medium, and strains were grown overnight either with appropriate CuSO_4_ concentrations to get steady-state induction at the same mean level (10 µM CuSO4 for *pCUP1_S__288__c_*-*ZeoR* and 5 µM for *pCUP1_S__288__c_-ZeoR*) or in the absence of CuSO_4_.

After dilution to OD_600_ 0.2, cultures were grown 5 h in the same culture conditions as overnight (either with the same CuSO_4_ concentrations or in the absence of CuSO_4_) prior to phleomycin exposure. Then, these cultures were diluted 100 times with the same media and divided into 11 aliquots. An appropriate volume of phleomycin solution (Invivogen) was added to generate a series of cultures containing 0–100 μg ml^−^^1^ phleomycin. This series was inoculated at 30 °C with 200 rpm shaking and the OD of each tube was measured after 24 h. For experimental growth time course at 40, 50, and 60 μg ml^−^^1^ phleomycin or without phleomycin, OD were followed during 35 h to draw the growth curve. All these experiments were repeated at least three times.

### Sequencing and Bioinformatics Analysis

Plasmids from 97 individual clones harboring noisy yEGFP expression were extracted using a standard phenol–chloroform extraction method and inserted fragments were sequenced using a primer hybridizing in the *yEGFP* gene 75 bp upstream of the start codon (supplementary table S7, Supplementary Material online). Sequencing was performed using an Applied Biosystems 3730xl DNA Analyzer. Base calling was performed using TraceTuner 3.0.4 beta (Denisov et al. 2004) to obtain fasta and quality values. Vector and quality trimming were performed using Lucy 1.19p (Chou and Holmes 2001). Only 96 reads were retained after trimming. Mapping reads was performed using SMALT 0.7.3 (http://www.sanger.ac.uk/resources/software/smalt/, last accessed March 23, 2015) resulting in 95 reads correctly mapped to the S288c genome. Variants were obtained using the mpileup command of SAMtools 0.1.18 (Li et al. 2009) and further filtered to keep those found upstream of ORF using a custom perl script. Gene Ontology (GO) analysis was performed using the *Saccharomyces* Genome Database Gene Ontology Term Finder. Sequence alignments were performed using the MultAlin software.

## Results

### Screening for High Noise Promoters in the EC1118 Industrial Yeast Genome

The genomic approach used to screen promoters conferring high promoter-mediated noise in the sequenced *S. cerevisiae* strain EC1118 (Novo et al. 2009) was based on the method developed initially with *Salmonella typhimurium* (Freed et al. 2008). In this modified protocol (see Materials and Methods), genomic DNA fragments from the haploid 59A strain (derivative of the winemaking diploid EC1118 strain) were inserted before the start codon of yEGFP in a series of three distinct promoterless yEGFP-coding vectors (the different plasmids contain zero, one or two additional base[s] between the insertion site and the *yEGFP* start codon). These centromeric plasmids minimized problems of copy number that would contribute to variations in fluorescence levels. The resulting library was transformed in the laboratory strain CEN.PK as we were looking for *cis*-effects on noise in yEGFP expression.

**Fig. 1.—:**
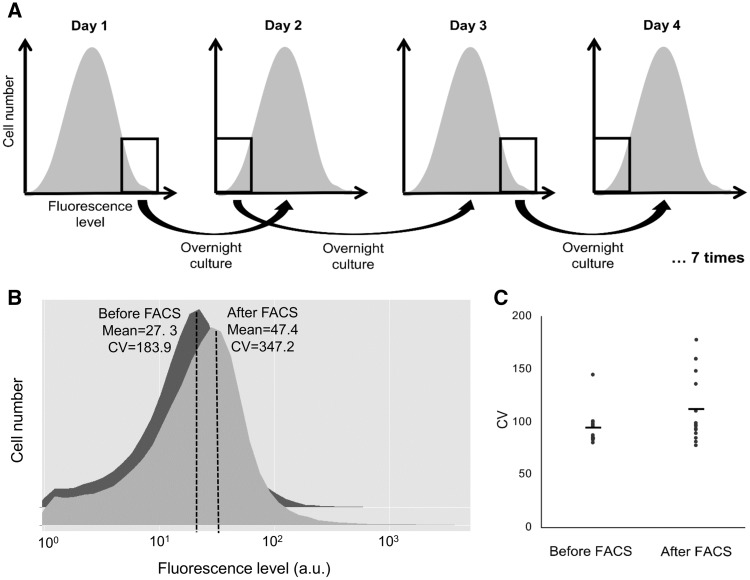
Fluctuating selection enriched for genomic fragments driving noisy yEGFP expression. (*A*) After insertion of genomic fragments from the EC1118 haploid derivative 59A into centromeric promoterless yEGFP-coding plasmids and transformation of the library in the *Saccharomyces cerevisiae* strain CEN-PK, fluctuating selection ensuring enrichment in noisy promoters was performed by seven rounds of cell sorting, with alternatively the 5% highest or the 5% lowest fluorescence levels conserved (except for the first round where only the fluorescent cells were sorted [around 4%]). Between each round, cells were grown overnight, diluted the next morning, and sorted in exponential phase in the afternoon. (*B*) Fluorescence distribution in the population containing the yEGFP-fused genomic library before and after fluctuating selection. Mean and CV values are increased after selection. (*C*) CV of yEGFP expression in clones from selected and control populations. The clones from the selected population (right) show higher noise level than the clones from the control population (left) (*T*-test, *P* = 0.047). Each data point represents the CV of yEGFP expression among several thousand individual cells.

Although no fluorescence was detected without genomic library, around 4% of the cells transformed with the library were fluorescent before any cell sorting (supplementary fig. S1, Supplementary Material online). Fluorescence levels above the autofluorescence threshold were spread along at least two logs, showing strong promoter activity of some fragments. The fluctuating selection method described by Freed et al. (2008) enabling enrichment of fragments producing highly variable yEGFP expression was then applied. Briefly, seven rounds of cell sorting were performed with alternatively the highest 5% or the lowest 5% fluorescence levels conserved (fig. 1*A*) (except for the first round where only the fluorescent cells were sorted). 10^5^ cells were sorted at each round, cultured overnight, diluted in the next morning to sort cells again in exponential phase in the afternoon at the same OD at each round. During the selection process, we chose for convenience to characterize cell–cell variability by the CV (standard deviation divided by the mean). As expected, the selected population exhibited higher CV: It approximately doubled after the selection procedure (fig. 1*B*). The mean expression level was also increased, showing that the library was enriched in fragments with promoter activity.

We also measured the CV of individual clones randomly isolated from the library before or after selection. Among the selected population, more clones showed high CV (*P* = 0.047) (fig. 1*C*). Thus, the fluctuating selection efficiently enriched the population in fragments giving high variability in yEGFP expression. Nevertheless, clones with noise levels similar to control clones were still present, as was previously observed with the original protocol (Freed et al. 2008) and might be explained by aggregation with nonfluorescent cells in the sorting process. Even if this method might not be the ideal way to strongly enrich for increased noise, selecting a smaller percentage of cells at each round could make the enrichment process more efficient. Here to avoid sequencing of “nonnoisy” clones, we screened for individual clones with high CV in the enriched library. By setting threshold values on mean and noise, only clones exhibiting highly variable yEGFP expression in the population were selected (supplementary fig. S2, Supplementary Material online). Expression profiles among these single clones were highly heterogeneous, with around 30% exhibiting bimodal expression (supplementary fig. S3, Supplementary Material online), but they all possessed CV among the highest CV values that are observed in the yeast genome (Newman et al. 2006), confirming the efficiency of the screening.

To confirm that phenotypic noise is a stable property of a clone, we reisolated on plates and analyzed two subclones from each selected clone. Ninety-nine clones with high CV were chosen for further investigations because mean and CV values of their subclones were highly reproducible compared with the initial clone (*R* > 0.97 for mean values and *R* > 0.8 for noise values) (supplementary table S1, Supplementary Material online). We also wanted to verify that the clones were mainly dominated by intrinsic noise originating from transcription. The contribution of extrinsic noise in total noise can be decreased by reducing the FSC and SSC gates (Newman et al. 2006). Indeed, although ungated populations are dominated by extrinsic noise, analysis on a more homogeneous part of the population decreases extrinsic noise either to levels comparable to intrinsic noise or to a level below that of intrinsic noise because the CV in GFP expression is calculated from a subset of cells similar in size, shape, and cellular complexity. Thus, to reduce in our CV measurements the extrinsic noise linked to cell-to-cell variations in global physiological factors, we extracted a subset of cells that were very homogeneous in size and granularity. By measuring fluorescence only on a more homogeneous part of the population (around 50% of the cells), CV were reduced by around 28.5% (supplementary table S2, Supplementary Material online). We concluded that promoter-mediated noise was the main contributor to the elevated CV observed in these clones.

### Genetic Differences between Noisy Promoters from EC1118 and Their Counterpart in S288c

The fragments driving yEGFP expression were successfully sequenced in 97 clones (table 1) and 95 were localized by mapping reads to the S288c reference genome (supplementary table S3, Supplementary Material online). First, around 33% of the inserted fragments were found at least two times, sometimes with different end points. These fragments with different ends were independently selected and reinforced the validation of the fluctuating selection. Second, the mean length of the fragments was around 650 bp. Most of them were fully sequenced by a single round of Sanger sequencing starting from 75 bp downstream the start codon of *yEGFP*. This mean length seemed low compared with the range of size selected to construct the genomic library (500–3,000 bp) and probably reflected the preference for smaller fragments in the cloning process. (Nevertheless this mean was slightly underestimated because a small minority of longer fragments has not been fully sequenced.) Third, the majority of fragments corresponded to promoter sequences (supplementary table S3, Supplementary Material online). A total of 50 distinct promoters were found (table 1) (a fragment is considered a promoter if its last base is at less than 350 bp from the start codon of the downstream ORF). Among these fragments with known promoter sequences, 27 contained the promoter only, with from 4 to 350 bp lacking before the start codon of the corresponding ORF, and 23 contained the promoter and a part of the corresponding ORF fused to *yEGFP* (supplementary table S3, Supplementary Material online). GO processes analysis on these 50 promoters revealed overrepresentation of genes involved in nitrogen compound transport (*P* = 0.04) and anion transport (*P* = 0.02). Plasma-membrane transporters are known to show significantly elevated expression noise (Zhang et al. 2009). Moreover, ion transport and nitrogen compound metabolic processes are among the few GOs identified to have greater-than-expected expression noise in yeast (Zhang et al. 2009).

**Table 1:** Summary of Sequencing Results and Mapping of Genomic Fragments from Clones Exhibiting Noisy yEGFP Expression

Reads and Genetic Differences	Number
Reads	97
Empty vectors	0
Mapped reads (to S288c)	95
Independent loci^a^	65
Fragments with known promoters	50
Known promoters with SNPs or INDELs compared with S288c	37
Total SNPs and INDELs in known promoters compared with S288c^b^	170

^a^The number of loci corresponds to independent genomic regions. Each locus might be found several times, sometimes with different ends (supplementary table S1, Supplementary Material online).

^b^The number of SNPs and INDELs is the total number of genetic differences in all the fragments identified as promoters between the S228c and EC1118 sequences.

We compared these EC1118 genomic fragments with their counterpart in S288c. A total of 170 genetic variations were detected in 37 of the 50 fragments containing known promoters (table 1, details in supplementary table S4, Supplementary Material online). The remaining 13 fragments did not show any difference between the strains. The variations were mostly SNPs and small INDELs ranging from 1 to 7 bp. Only one longer insertion of 21 bp was present in a promoter from EC1118. The number of variations greatly varied from promoter to promoter, ranging from 1 SNP to 15 different variations including SNPs and INDELs. Interestingly promoters driving expression of genes involved in stress response (e.g., *HAC1*, *CUP1*) or in diverse transports (e.g., *CAN1*, *GNP1*, *AGP2*) were present in this list. We hypothesized that some of these natural genetic variations could generate differences in noise level in the expression of these genes, and thus could confer an adaptive advantage in specific challenging environments. Eight promoters were particularly interesting because they are related to environmental factors (table 2). We choose these genes on the basis of their function and not depending on either the nature or the localization of the genetic variations because even the effects of mutations in regions well-known to modify noise strongly depends on promoter context (Hornung et al. 2012) and because of the high number of potential binding sites for transcription factors in these promoters.

**Table 2:** Genes Whose Promoter Variants Are Studied at the Genomic Level

Gene	Function
BMH1/YER177W	14-3-3 protein, major isoform, regulates many processes
BMH2/YDR099W	14-3-3 protein, minor isoform, regulates many processes
CUP1/YHR055C	Binds copper, mediates resistance to high concentrations of copper
CAN1/YEL063C	Plasma membrane arginine permease
YCK2/YNL154C	Palmitoylated plasma membrane-bound casein kinase I isoform
HAC1/YFL031W	Transcription factor, regulates the unfolded protein response
GNP1/YDR508C	High-affinity glutamine permease; also transports Leu, Ser, Thr, Cys, Met, Asn
AGP2/YBR132C	Plasma membrane regulator of polyamine and carnitine transport

### Noise Levels Conferred by Each Variant of Selected Promoters at the Genomic Level

We decided to finely compare at the genomic level the expression and noise levels conferred by both variants of these promoters of interest. Indeed, plasmids do not fully recapitulate chromosomal organization and might generate experimental bias. It was also necessary to compare expression profiles from each variant in both strains (BY4720, an auxotrophic derivative of S228c, and 59A, the haploid derivative of EC1118) to distinguish *cis*-effects (promoter variations) on noise from *trans*-effects linked to the genetic background. Indeed, strain effects on noise are known (Ansel et al. 2008) and require determining whether epistasis is observed in the generation of promoter-mediated noise. Comparing promoter pairs in both strains also required insertion of the variants in strictly the same chromosomal context. We took advantage of an insertion plasmid (pJRL2) previously used to compare expression variability from mutated *PHO5* promoters inserted in the *LEU2* locus (Raser and O*’*Shea 2004). By replacing the *PHO5* promoter with our 16 variants, we were able to insert them in the same locus. We chose to clone 1,000 bp before the start codon, and not only the fragment inserted in the library plasmids. These longer loci sometimes contained more genetic variations between S228c and EC1118 compared with the cloned fragments but did not contain any additional gene or promoter.

To be as accurate as possible, we wanted to reduce extrinsic noise even if both variants of the same promoter were always compared in a given genetic background. Thus, fluorescence levels were measured in small gates where cells are homogenous in terms of size and granularity (strictly the same gate was used for the different variants of a promoter in a given background). Also, noise was calculated in the next steps by dividing the variance by the squared mean because this value reflects better than the CV how large the standard deviation is compared with the mean expression level (Chalancon et al. 2012). Mean expression and noise levels have been measured in optimal growth conditions and exponential phase at the same OD. Finally, the 59A strain possesses a high aggregation tendency making flow cytometry analysis difficult. Therefore, the *amn1Δ* 59A strain was used because Amn1p plays the leading role in this cell aggregation (Li et al. 2013).

**Fig. 2.—:**
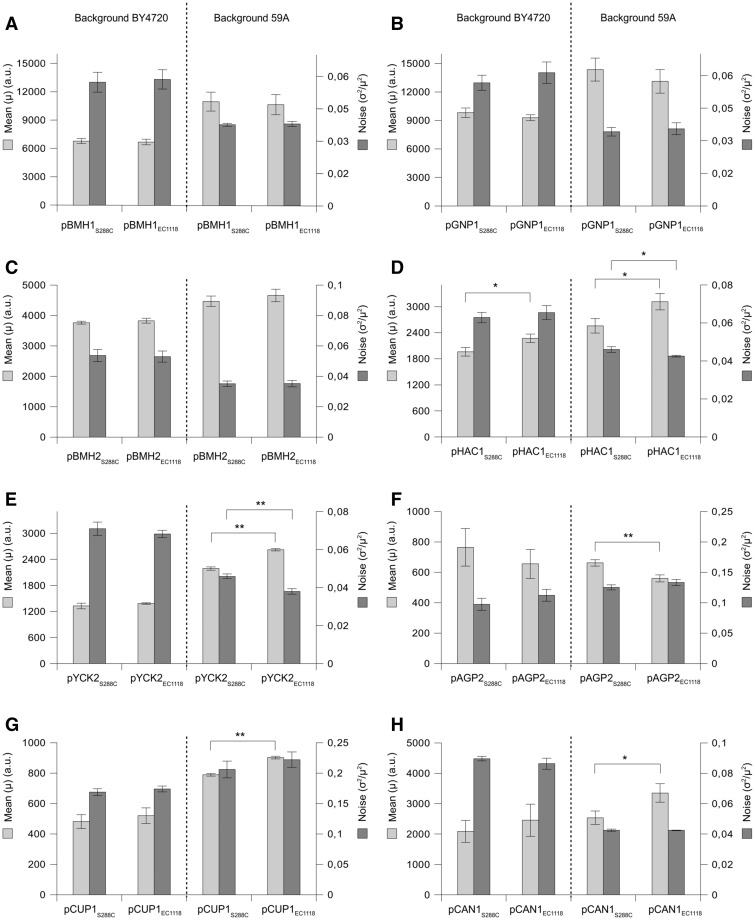
Different behaviors of promoter variants at the genomic level. Mean and noise values of yEGFP expression conferred by each variant of eight promoters were measured at the genomic level (*LEU2* locus) in the BY4720 (auxotrophic derivative of S288c) and 59A (haploid derivative of EC1118) strains. Dashed lines separate the results obtained in the different backgrounds for a given promoter pair (BY4720 on the left, 59A on the right). (*A–H*) Results for the *pBMH1, pGNP1, pBMH2, pHAC1, pYCK2, pAGP2, pCUP1,* and *pCAN1* promoter variants, respectively. Scale for the mean is on the right and scale for the noise is on the left of the histograms. The same nomenclature is used for each histogram: Each variant is named by the promoter name with the name of the strain where it comes from in subscript (S288c or EC1118). Results are means of three independent cultures, and error bars are standard deviations. A significant statistical difference between mean or noise levels conferred by the promoter variants in a given genetic background is represented by (*) when *P* < 0.05 in *T*-test or (**) when *P* < 0.01.

The results obtained with the same promoter pair in both strains are presented on the same histogram in figure 2 but it is worth noting that comparing results for a given variant in the different backgrounds is not possible because the gates where fluorescence levels were measured were not strictly identical in both strains (59A had higher cell size and granularity). Nevertheless, as we compared the consequences on noise of promoter sequence variations and their dependence on the *trans*-background, we chose to show them on the same plot.

On one hand, both variants of *pBMH1*, *pGNP1,* and *pBMH2* gave the same mean expression and noise levels in each strain (fig. 2*A*–*C*). The genetic differences between these variants did not generate any effect on fluorescence profiles. On the other hand, the five other promoter pairs exhibited differences in mean and/or noise in at least one background. *pHAC1_EC__1118_* conferred higher mean expression compared with the lab variant in both backgrounds (*P* = 0.02 and *P* = 0.018) (fig. 2*D*), whereas *pYCK2_EC__1118_*, *pCUP1_EC__1118_*, and *pCAN1_EC__1118_* gave increased expression only in 59A (*P* = 10^−^^4^, *P* = 10^−^^4^, and *P* = 0.02, respectively) (fig. 2*E*, *G*, and *H*), indicating a strain-effect that contributed to reveal the consequences of the genetic variations between the variants. This was also observed with *pAGP2* but here expression was lower with the industrial variant only in 59A (*P* = 0.005) (fig. 2*F*). A higher expression for one version was associated with lower noise only in 2/6 cases among these promoter pairs whereas higher mean expression is generally accompanied by decreased expression variability (Bar-Even et al. 2006; Newman et al. 2006; Hornung et al. 2012; Carey et al. 2013). Thus, the laboratory and industrial variants of these promoters differed by their mean expression in at least one background, but enhanced expression did not necessarily produce lower noise so that the genetic variations might generate a different level of noise at similar mean expression level. This hypothesis was tested by inducing the *pCUP1* variants with different copper concentrations.

### Noise Levels Conferred by the *pCUP1* Variants during Copper Induction

*CUP1* is involved in copper detoxification. The *CUP1* copy number is highly correlated to copper resistance (Fogel and Welch 1982) and strains evolving in copper-rich environments amplify *CUP1* and contain many copies (Adamo et al. 2012; Chang et al. 2013). Moreover, its promoter has been studied in detail for instance in terms of transcription factors binding kinetics (Karpova et al. 2008) or nucleosome repositioning (Shen et al. 2001) during copper induction. We induced the *pCUP1* variants by copper to determine under which conditions mean expression levels were similar with both variants, and whether increased noise was conferred by *pCUP1_EC__1118_* in this case.

**Fig. 3.—:**
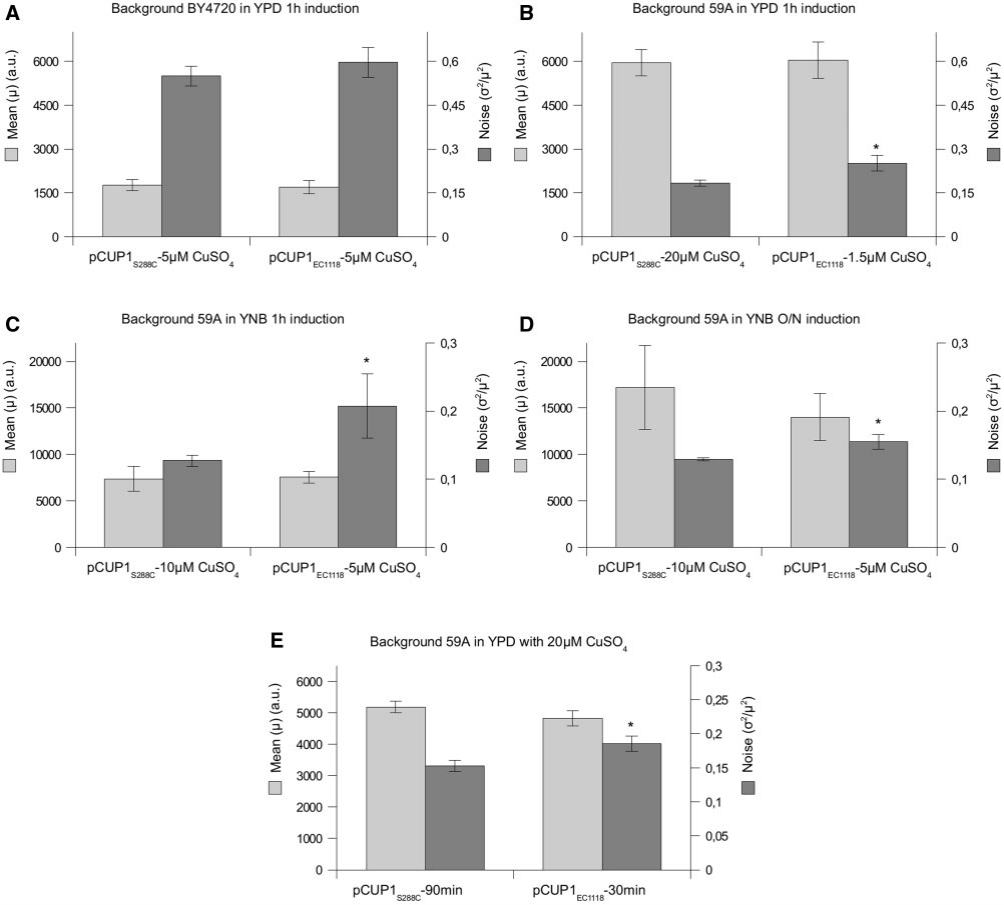
The *pCUP1_EC1118_* promoter variant is noisier than *pCUP1_S288c_* at equal mean expression level in 59A. Mean and noise values of yEGFP expression conferred by each variant of the *CUP1* promoter were measured in different conditions enabling comparison of noise levels independently of the mean. (*A*) In the BY4720 background and in YPD medium after 1-h induction by 5 µM CuSO_4_ for *pCUP1_S288c_* and *pCUP1_EC1118_*. (*B*) In the 59A background and in YPD medium after 1-h induction by 20 and 1.5 µM CuSO_4_ for *pCUP1_S288c_* and *pCUP1_EC1118_*, respectively. (*C*) In the 59A background and in YNB medium after 1-h induction by 10 and 5 µM CuSO_4_ for *pCUP1_S288c_* and *pCUP1_EC1118_*, respectively. (*D*) In the 59A background and in YNB medium after overnight induction by 10 and 5 µM CuSO_4_ for *pCUP1_S288c_* and *pCUP1_EC1118_*, respectively. (*E*) In the 59A background and in YPD medium with 20 µM CuSO_4_ after 1 h 30 min induction for *pCUP1_S288c_* and 30-min induction for *pCUP1_EC1118_*. Data are means of three independent cultures, and error bars are standard deviations. A significant statistical difference between noise levels conferred by the promoter variants is represented by (*) when *P* < 0.05 in *T*-test.

We first measured mean expression levels after 1-h induction when cells were exposed to different copper sulfate (CuSO_4_) concentrations in YPD medium (supplementary fig. S4*A* and *B*, Supplementary Material online). In both strains and with both variants, induction increased with copper sulfate concentration to reach a plateau in concentrations higher than 20 µM CuSO_4_ in BY4720, and 6 µM in 59A. Although the variants behaved very similarly in BY4720 (supplementary fig. S4*A*, Supplementary Material online), *pCUP1_EC__1118_* was more strongly induced than *pCUP1_S__288__c_* in 59A at each concentration (supplementary fig. S4*B*, Supplementary Material online). We chose 5 µM CuSO_4_ for both variants in BY4720, and 20 and 1.5 µM CuSO_4_ for *pCUP1_S__288__c_* and *pCUP1_EC__1118_*, respectively, in 59A to compare noise at similar mean levels and thus independently of the mean. These concentrations avoided experimental bias linked to heterogeneous copper concentrations in the basis medium. We found no differences in BY4720 (*P* = 0.25) (fig. 3*A*) but *pCUP1_EC__1118_* was clearly noisier than *pCUP1_S__288__c_* in 59A (*P* = 0.015) (fig. 3*B*). Of note, this difference was also observed in YNB medium after 1-h induction and overnight induction (*P* = 0.045 and *P* = 0.016, respectively) (fig. 3*C* and *D*) (see supplementary fig. S4*C* and *D*, Supplementary Material online, for dose–response curves). Finally, the difference in noise was still observed in the same CuSO4 concentration (10 µM) in YNB medium after 1-h induction (*P* = 0.02) (mean values are not significantly different in this case whereas *pCUP1_EC__1118_* conferred a slightly higher mean [*P* = 0.12]) (supplementary fig. S5, Supplementary Material online). Therefore, this natural variant of *pCUP1* exhibited higher promoter-mediated noise in gene expression.

We also searched for induction times giving the same mean expression for both variants in each strain when cells were exposed to 20 µM CuSO_4_ in YPD medium. On one hand, induction curves were similar for both variants in BY4720 (supplementary fig. S4*E*, Supplementary Material online). It increased in the first hour of induction and then decreased in the next 1.5 h. On the other hand, the promoters’ behavior is different in 59A: Induction was clearly stronger with *pCUP1_EC__1118_* (supplementary fig. S4*F*, Supplementary Material online). At similar mean expression levels (*t* = 30 min for *pCUP1_EC__1118_* and *t* = 90 min for *pCUP1_S__288__c_*), *pCUP1_EC__1118_* was clearly noisier than *pCUP1_S__288__c_* (fig. 3*E*) (*P* = 0.014). In spite of the slightly lower mean expression from the industrial variant that could favor the observed difference in noise in figure 3*D* and *E*, these results were in the same tendency as results in figure 3*B* and *C* giving a statistically significant difference in noise with very similar mean levels. Also, increased noise is still observed with *pCUP1_EC__1118_* at the same copper concentration in YNB medium (supplementary fig. S5, Supplementary Material online), even if its mean value is slightly higher (while mean values are not significantly different), so that *pCUP1_EC__1118_* appears to be clearly noisier in all conditions. Finally, it is worth noting that the induction factor is far higher in 59A than in BY4720 for both variants (about 5–7 and 1.5, respectively, in 20 µM CuSO_4_).

Various promoter elements contribute to noise mainly by modulating mRNA production burst size and frequency and thus noise (Sanchez et al. 2013; Sanchez and Golding 2013). Here, three SNPs and one 4-base deletion exist in *pCUP1_EC__1118_* compared with *pCUP1_S__288__c_* (supplementary fig. S6, Supplementary Material online). Of note, these variations are common in ten other wine strains whereas they are generally not present in laboratory strains (except in sigma1278b) (supplementary fig. S6, Supplementary Material online). Other SNPs and INDELs are also commonly found in *pCUP1* in wine strains whereas the coding sequence is always identical either in lab or in wine strains (the SNP in T73 is synonymous), showing that the *CUP1* transcription kinetics might be subject to many changes due to *cis*-modifications of the *CUP1* promoter. The SNPs between *pCUP1_EC__1118_* and *pCUP1_S__288__c_* are upstream of the transcription starting site, and the deletion is in the 5′-UTR. Several transcription factor binding sites are suppressed in *pCUP1_EC__1118_*, but only in the reverse orientation (supplementary table S5, Supplementary Material online). The first SNP is in an HSF1p binding site, but modifies a position where any nucleotide can be found. We performed directed mutagenesis on each SNP or INDEL position in *pCUP1_S__288__c_* and measured mean and noise in 59A with induction at 5 µM CuSO_4_. Only the second SNP conferred significantly higher mean expression compared with *pCUP1_S__288__c_* (supplementary fig. S7, Supplementary Material online). Nevertheless, no significant change in noise level was observed so that this SNP might contribute to increase cell–cell variability at equal mean expression levels.

### Benefit Conferred by the Noisiest *pCUP1* Promoter Variant

**Fig. 4.—:**
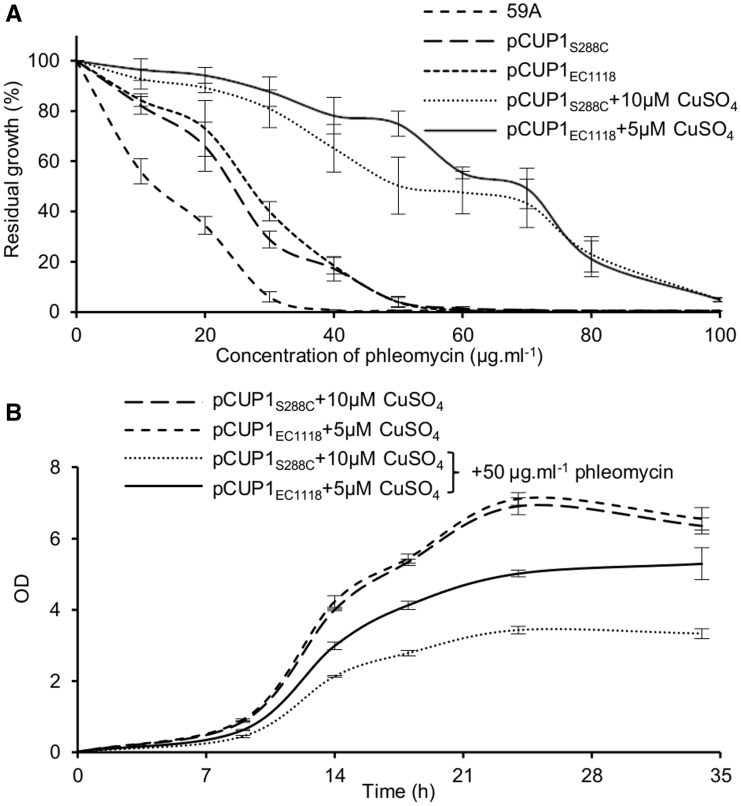
The *pCUP1_EC1118_* and *pCUP1_S288c_* promoter variants confer distinct abilities to survive in a selective environment. Phenotypic consequences of the different noise levels conferred by the *pCUP1_S288c_* and *pCUP1_EC1118_* variants fused to *ZeoR* were measured in phleomycin-containing medium. (*A*) Residual growth after 24-h treatment with various phleomycin concentrations. Residual growth was determined by normalizing OD600 measurement for cultures grown in the presence of phleomycin to an identical dilution grown the absence of phleomycin. Strains were previously induced overnight with appropriate CuSO_4_ concentrations to make the strains express *ZeoR* at similar mean expression levels. CuSO_4_ was maintained during phleomycin treatment. Data points are the means from three independent cultures at 24-h growth in the presence of indicated amounts of phleomycin, and error bars are standard deviations. (*B*) Experimental growth time course without phleomycin or at 50 µg/ml phleomycin with appropriate CuSO_4_ concentrations to make the strains express *ZeoR* at similar mean expression levels. Data points are means of three independent cultures, and error bars are standard deviations.

It remained to consider whether this higher noise observed with *pCUP1_EC__1118_* might confer a benefit and increase population survival upon exposure to constant selective conditions. To test for selective advantages, each variant was fused to the She ble gene (*ZeoR*) (conferring resistance to phleomycin) in the pJRL2 plasmid and integrated in the *LEU2* locus in 59A. The main problem with using copper as a selective agent in these growth experiments would have been the need to use the same copper concentration for both variants. Indeed, as they are not induced in the same manner by copper (less copper is needed to get the same mean expression with the industrial variant), we would have had differences of mean expression levels between the variants by using copper as a selective agent, making interpretations about the impact of noise impossible. This led us to choose *ZeoR* and phleomycin as an adequate system to test our hypothesis. Experiments were performed at steady-state induction levels after overnight induction (see Materials and Methods and Blake et al. 2006). Briefly, each strain was induced in adequate copper concentrations to obtain similar mean expression levels and differences in noise in YNB medium. In exponential phase, each strain was or was not exposed to different concentrations of phleomycin to determine the residual growth at each concentration (fig. 4*A*). Although the 59A control without *ZeoR* was highly sensitive, induced strains had the highest residual growth and noninduced strains had intermediate phenotypes. *pCUP1_EC__1118_* conferred a slightly higher residual growth in 30 µg/ml phleomycin without copper, probably linked to the higher basal mean expression (fig. 2*G*). In inducing conditions, *pCUP1_EC__1118_* significantly improved growth in 50 µg/ml phleomycin compared with *pCUP1_S__288__c_* (fig. 4*A*). In lower and higher concentrations, both strains exhibited the same behavior. Thus, the effects of the increased cell–cell variability conferred by this variant on growth in selective environment are observed only in a specific range of phleomycin concentrations.

To finely determine growth kinetics, using flow cytometry, we followed the growth curves in 40, 50, and 60 µg/ml phleomycin in proper copper concentrations for both strains to have identical mean expression levels, as well as the growth curves with copper only. Although growth did not show any difference in 5 and 10 µM CuSO_4_ without phleomycin, *pCUP1_EC__1118_*-*ZeoR* induced in 5 µM CuSO_4_ gave a better growth than *pCUP1_S__288__c_*-*ZeoR* induced in 10 µM CuSO_4_ at all measurement points in 50 µg/ml phleomycin (fig. 4*B*). We also observed a significant difference in 40 and 60 µg/ml phleomycin (supplementary fig. S8, Supplementary Material online) but it was less important, as one could have expected by looking at the residual growth curves (fig. 4*A*). Taken together, this result confirmed that the difference of promoter-mediated noise between the natural promoter variants *pCUP1_EC__1118_* and *pCUP1_S__288__c_* is sufficient to confer different abilities to survive, but only in a given range of selective pressure.

## Discussion

The genetic determinants of transcriptional-mediated noise have been characterized by rationally manipulating or randomly mutating gene promoters (Sanchez and Golding 2013). All these studies unravel the origins that underlie gene-specific expression variability by showing that intrinsic noise is at least in part generated by *cis*-acting regulatory elements embedded within the DNA sequence of each promoter. As far as we are aware, there are no studies focused on natural promoter variants that may confer different noise levels and consequently different abilities to survive in stressful environments, so that the relevance to adaptation in natural systems remains to be determined (Ackermann 2013; Viney and Reece 2013; Holland et al. 2014).

Here, we screened for promoters conferring high noise in the genome of the haploid 59A yeast strain derived from the industrial wine EC1118 strain to search for natural variants conferring adaptive advantage in stressful conditions through enhanced cell–cell variability. As expected, sequenced EC1118 genomic fragments conferring noisy expression were enriched in GO categories possessing significantly greater-than-expected expression noise (Zhang et al. 2009). Especially, promoters of genes involved in nitrogen compounds transport were overrepresented. Genes implicated in nitrogen metabolism are also among the genes showing significant variation in expression among natural isolates of *S. cerevisiae* (Carreto et al. 2011). These data should make sense because a positive correlation is known between gene expression noise and gene expression divergence in yeast (Lehner 2008; Zhang et al. 2009). Moreover, nitrogen assimilation is highly variable among wine strains and correlates to fermentation efficiency (Treu et al. 2014).

Our study of eight promoter variant pairs at the genomic level reveals that higher mean expression with one variant was not always associated with lower noise while this correlation has been reported many times (Newman et al. 2006; Hornung et al. 2012; Carey et al. 2013). When differences in mean expression exist, higher expression is generally observed with promoters from EC1118 (except for *pAGP2*). Nevertheless, it often depends on the genetic background because the variants of some promoters give different mean expression levels in 59A whereas they do not show any difference in BY4720. Therefore variations in *cis*- (promoter sequence) and *trans*- (cellular factors involved in gene expression) factors are associated to enable this enhanced expression in the industrial strain. Interestingly, the difference in noise observed between variants of several promoter pairs also depends on the genetic background, revealing epitasis in the generation of promoter-mediated noise.

We confirmed the difference in terms of noise between the *pCUP1* promoter variants by inducing them in different copper sulfate concentrations conferring the same mean expression level. One problem could be that changes in gene expression level could be from gene expression burst frequency or/and burst size and that adding more copper into a media might increase burst frequency and thus produce less gene expression noise for the laboratory variant. Nevertheless the difference in noise observed between the two variants in these induction conditions seems not to be due to changes in burst frequency generated by different copper concentrations because at the basal level, we already observe a nonexpected result between the two variants of the *CUP1* promoter in 59A (while it is not observed in S288c): The higher mean conferred by the industrial variant was not associated with lower noise. Moreover, *pCUP1_EC__1118_* is still noisier than pCUP1_S288c_ in 59A at the same copper concentration, even if the mean level conferred by *pCUP1_EC__1118_* is higher (while mean values are not significantly different in supplementary fig. S5, Supplementary Material online). Thus, increased noise is a feature of the industrial variant only revealed in the 59A background, independently of copper concentration. This result indicates that the genetic variations probably increase burst size and decrease burst frequency at the same copper concentration in 59A, explaining the increased mean and noise levels.

Differences in global constraints on noise have already been reported between yeast strains (Ansel et al. 2008; Fehrmann et al. 2013) but most studies showed that stochastic transcriptional kinetics in yeast is mainly determined by gene-specific effects (Sanchez et al. 2013). Here, we show the combined influences of *cis*- and *trans*-acting factors contributing to enhance expression noise for a given gene. Thus, as suggested recently, single-cell transcriptional kinetics are affected by both promoter architecture and genome-wide processes in yeast (Sanchez and Golding 2013). The hypothesis of noisier expression of regulatory factors (noise propagated in regulatory pathways; Blake et al. 2003) is not relevant because both versions would be identically affected. As *pCUP1_EC__1118_* enhances noise only in 59A, proteins involved in *CUP1* transcription or in the global transcriptional process in this strain might be more sensitive to genetic variations in *pCUP1* in terms of promoter binding. This hypothesis might provide an explanation for the epistatic interaction in the consequences of *pCUP1* sequence modifications. In any case, the consequences of the SNP or the deletion between the *pCUP1* variants are not dramatic and might reveal that natural systems evolve through promoter modifications generating small effects on noise, and not strongly affecting cell–cell variability such as the ones produced by rational manipulation of yeast promoters.

Phenotypic consequences of noise in gene expression in terms of survival in selective environments are little studied (Viney and Reece 2013). Only artificial systems using rationally modified promoters have been employed to test noise-mediated fitness differences (Blake et al. 2006; Smith et al. 2007). For instance, by introducing mutations within the TATA region of an engineered *S. cerevisiae GAL1* promoter, Blake et al. have shown that increased cell–cell variability in the expression of *ZeoR* confers a clear benefit in Zeocin containing medium (Blake et al. 2006). Nevertheless differences in noise between the mutated promoters were very high and this proof of principle did not imply that such an adaptation mechanism through noise modulation may naturally occur. By fusing natural yeast variants of *pCUP1* with *ZeoR*, we show here that their different noise levels at equal mean expression indeed provide distinct abilities to survive in constant environmental stress, even if the noise difference is far less important than in Blake’s study. Growth curves show that growth reduction is only observable in a specific range of phleomycin concentrations. This result is quite different from Blake’s study where enhanced noise was always either disadvantageous or beneficial in all tested concentrations. Here, a benefit is conferred by *pCUP1_EC__1118_* at intermediate concentrations of phleomycin and no growth difference with *pCUP1_S__288__c_* exists at lower or higher antibiotics concentrations. The slightly more heterogeneous expression distribution with *pCUP1_EC__1118_* might provide a possible explanation. Indeed, the difference between the distributions might be too weak to reveal a selective advantage at high phleomycin concentrations because very few cells express more ZeoRp with *pCUP1_EC__1118_* in the extreme subpopulation. On the contrary, at intermediate concentrations a larger proportion of the population is above the expression threshold necessary to grow in these concentrations. Therefore, the difference between the distributions might be sufficient in this case to generate a benefit because more cells express more ZeoRp with *pCUP1_EC__1118_*.

Identification of a stronger *pCUP1* in an industrial wine strain makes sense because wine yeasts are frequently exposed to high copper content in fermentation must, especially because the Bordeaux mixture containing copper sulfate is widely used as a fungicide in vineyards. *CUP1* is one of the best examples of high correlation between evolution in a stressful environment, the expression level of the gene conferring resistance to this environment, and the resistance itself (Adamo et al. 2012; Chang et al. 2013). One can also expect that the genome of wine strains would contain more *CUP1* copies than laboratory strains, which is actually not the case as for EC1118 as well as for other wine strains (Dunn et al. 2005). This might be explained by a selective disadvantage of having lots of *CUP1* copies counterbalancing the benefits of copper resistance. Interestingly, various genes involved in stress response, especially *CUP1*, have been positively correlated with the fermentation duration (Ambroset et al. 2011). Thus, a high stress response was associated with a low fermentation capacity. Evolution of *CUP1* toward higher noise in its expression is conceivable: High mean expression is both advantageous in high copper concentrations and disadvantageous for fermentation. These traits exert opposing selective pressures on *CUP1* expression. Noisier expression would be advantageous in that it would make the population harbor an optimum between fermentation capacity and adaptability to copper rich environments when necessary. In bacteria, a constant selective pressure selects for mutants harboring either higher mean with no noise increase or higher noise with similar mean (Ito et al. 2009). But opposing selective pressures would mainly select for increased expression variability. Thus, *pCUP1* might have evolved toward higher noise because it might increase fitness in fermentation-associated copper-rich environments, where high *CUP1* expression is advantageous regarding copper, and disadvantageous regarding fermentation efficiency. More direct evidence could be provided by evolving *S. cerevisiae* in controlled fermentative copper-rich environments. It would likely confirm the selection for noisy *CUP1* expression. It has recently been shown that environmental stress selects for organisms with increased phenotypic heterogeneity in yeast populations, but no links with increased variability in the expression of key genes have been established yet (Holland et al. 2014). Finally, numerous genomic studies are in progress and new yeast genomes will be available soon to determine whether positive selection seems to have recently occurred on the *pCUP1* of wine strains. Genetic studies would also determine whether the observed genetic variations are under positive selection or whether alternative explanations (neutral evolution, relaxed selective constrain, or fixation of slightly deleterious mutation) should be favored.

Collectively, our results provide evidence that natural yeast promoter variants can exhibit different levels of transcriptional-mediated noise and that epistasis exists in the generation of this noise: The combined influences of promoter sequence modifications and of the *trans*-background contribute to modify it. Finally, we show that natural yeast promoter variants conferring distinct abilities to survive in a stressful environment through noise modulation can be found among *S. cerevisiae* strains, showing that this possible adaptation mechanism has to be considered when studying yeast evolution and when exploring natural and artificial genetic diversity to improve industrial yeast strains (Steensels et al. 2014).

## Supplementary Material

Supplementary tables S1–S7 and figures S1–S8 are available at *Genome Biology and Evolution* online (http://www.gbe.oxfordjournals.org/).

Supplementary Data
