# Supplementary material for: Natural Yeast Promoter Variants Reveal Epistasis in the Generation of Transcriptional-Mediated Noise and Its Potential Benefit in Stressful Conditions
Source: Genome Biol Evol. 2015 Mar 11;7(4):969–84. doi: 10.1093/gbe/evv047 (PMC4419794; doi:10.1093/gbe/evv047)
Supplement: Supplementary Data [file supp_7_4_969__index.html]

Natural Yeast Promoter Variants Reveal Epistasis in the Generation of Transcriptional-Mediated Noise and Its Potential Benefit in Stressful Conditions — Supplementary Data 

# Natural Yeast Promoter Variants Reveal Epistasis in the Generation of Transcriptional-Mediated Noise and Its Potential Benefit in Stressful Conditions

## Supplementary Data

files

**Files in this Data Supplement:**

- Supplementary Data - pdf file
- Supplementary Data - xls file
- Supplementary Data - xls file
