# Supplementary material for: Natural Yeast Promoter Variants Reveal Epistasis in the Generation of Transcriptional-Mediated Noise and Its Potential Benefit in Stressful Conditions
Source: Genome Biol Evol. 2015 Mar 11;7(4):969–84. doi: 10.1093/gbe/evv047 (PMC4419794; doi:10.1093/gbe/evv047)
Supplement: Supplementary Data [file supp_evv047_Supplementary_material_revised_version.pdf]

Supplementary Material for:

Natural yeast promoter variants reveal epistasis in the generation of transcriptional-mediated noise and its potential benefit in stressful conditions

Jian Liu, Hélène Martin-Yken, Frédéric Bigey, Sylvie Dequin, Jean-Marie François & Jean-Pascal Capp

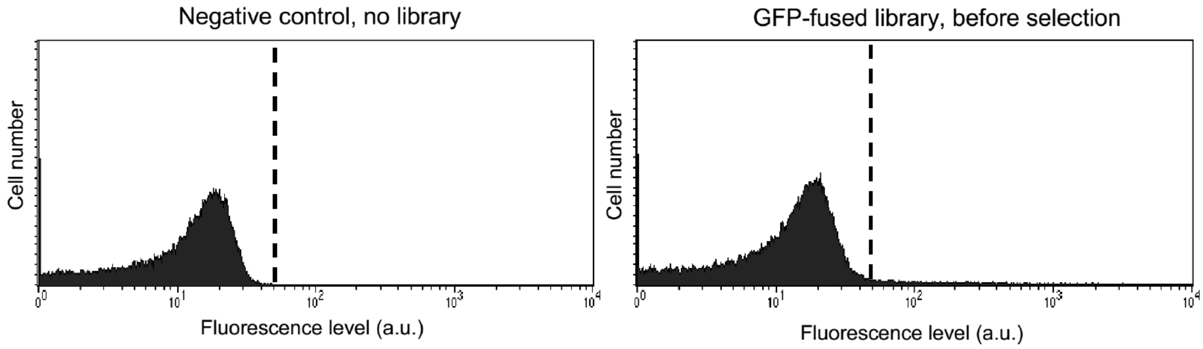

Supplementary Figure 1. Fluorescence distribution in the control population and the population transformed with the yEGFP-fused genomic library. After construction of the library by insertion of genomic fragments from the EC1118 strain in promoterless yEGFP-coding vectors and transformation of the library in the CEN.PK strain (on the right), around 4% of the population were above the auto-fluorescence threshold (dashed line) determined with the control population (on the left).

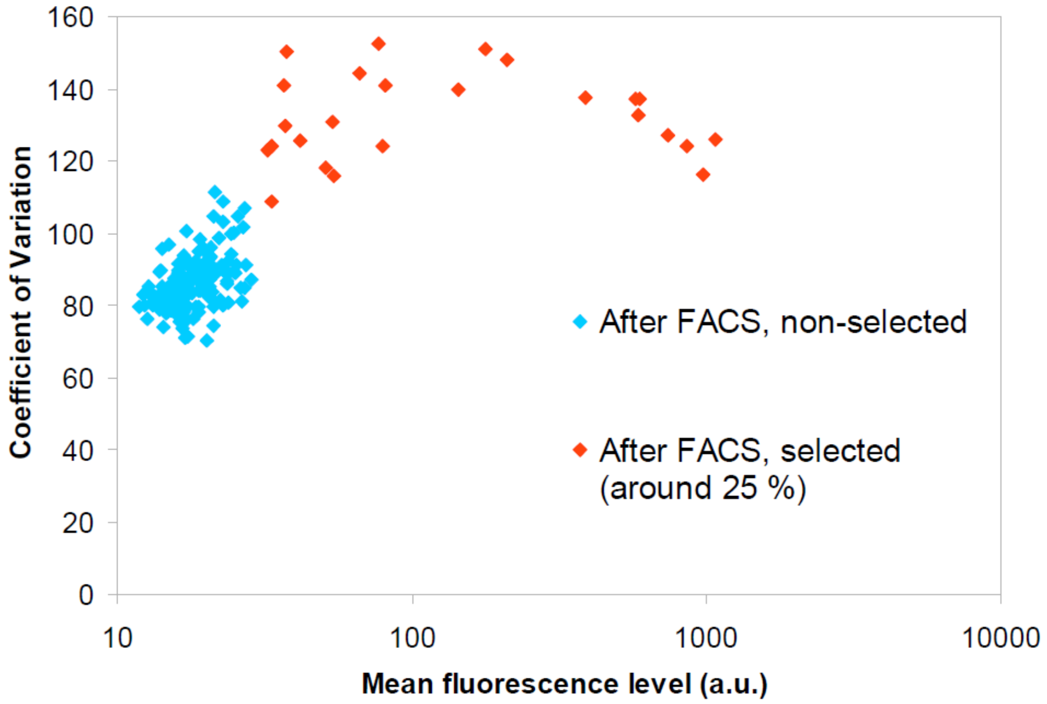

Supplementary Figure 2. Screening for clones with high CV among the selected population. Clones from the selected population with CV above 110% and mean expression above 30 arbitrary units were conserved for further studies.

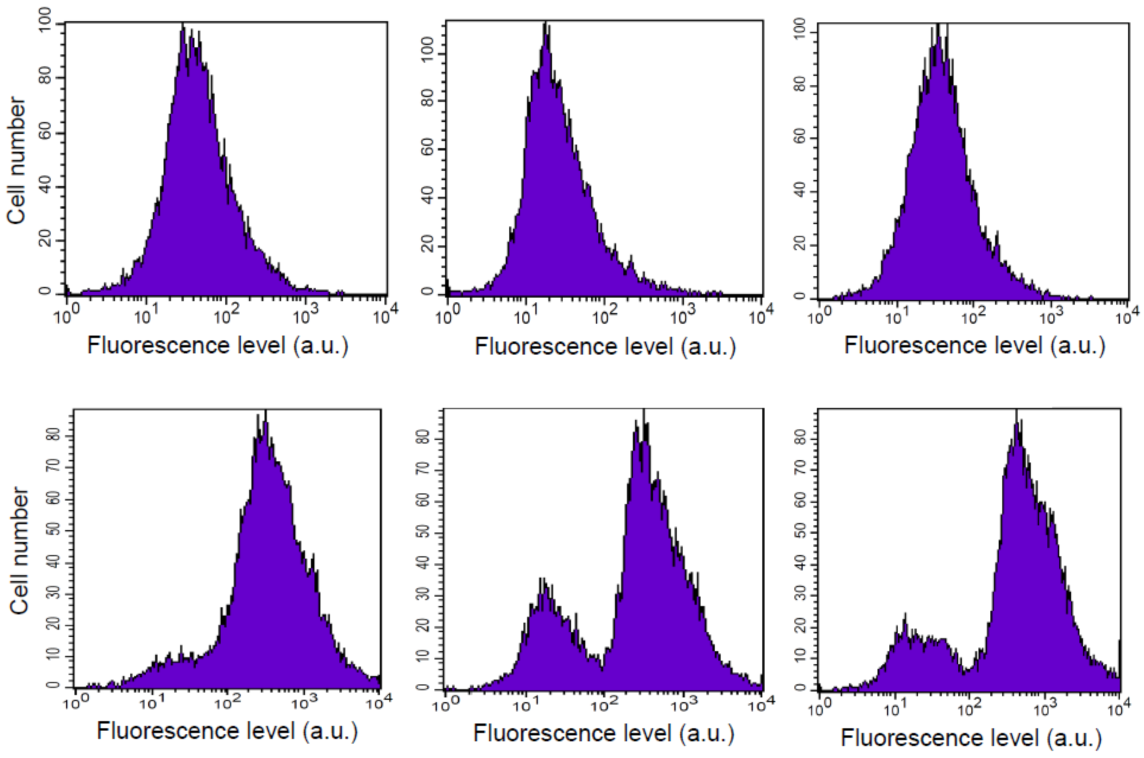

Supplementary Figure 3. Fluorescence distribution in some selected clones showing noisy yEGFP expression.

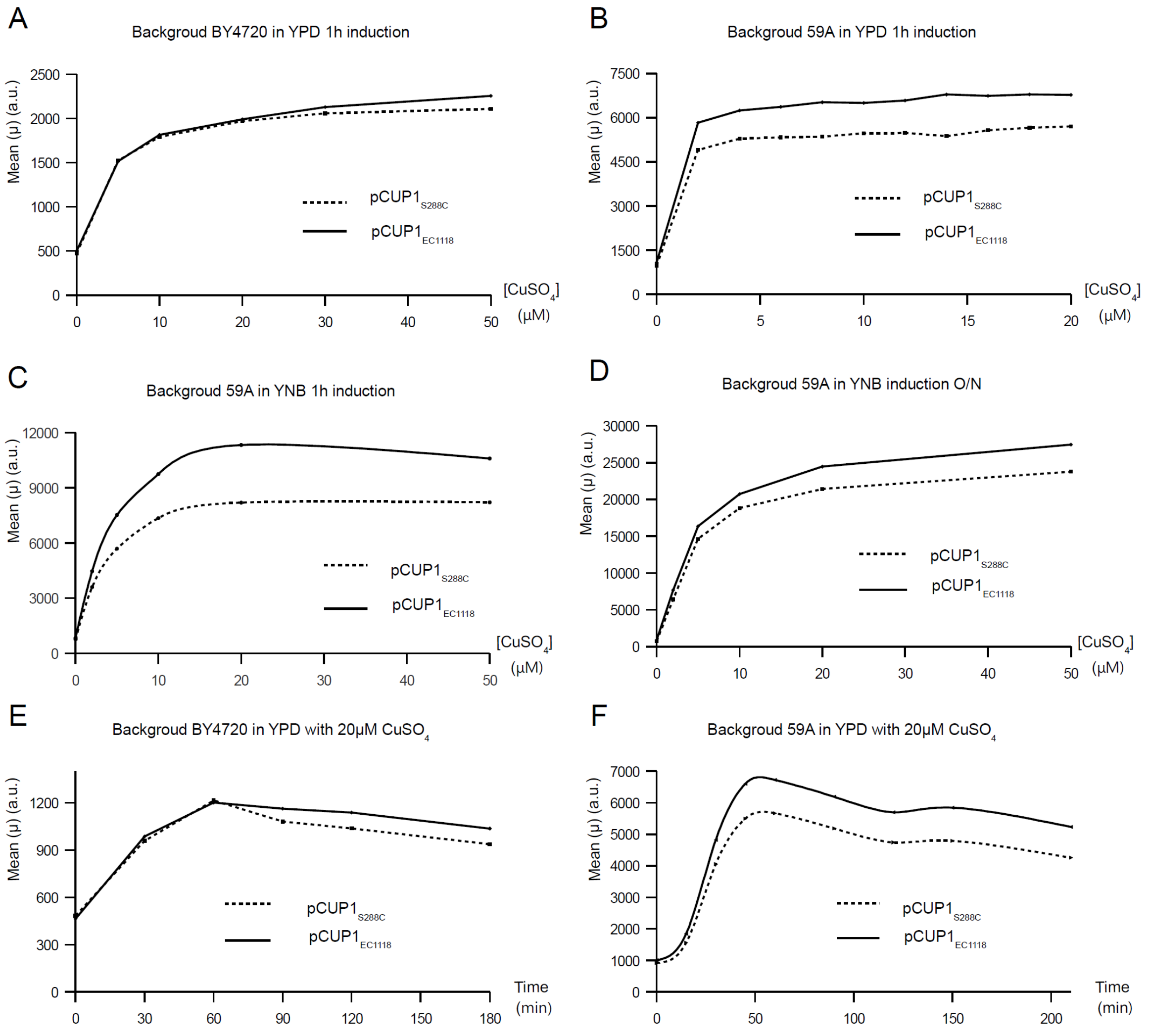

Supplementary Figure 4. Induction curves of the *pCUP1*$_{S288c}$ and *pCUP1*$_{EC1118}$ promoter variants. (A) In YPD medium, BY4720 background and 1h induction with varying $CuSO_4$ concentrations. (B) In YPD medium, 59A background and 1h induction with varying $CuSO_4$ concentrations. (C) In YNB medium, 59A background and 1h induction with varying $CuSO_4$ concentrations. (D) In YNB medium, 59A background and overnight induction with varying $CuSO_4$ concentrations. (E) In YPD medium, BY4720 background and 20μM $CuSO_4$ with varying induction times. (F) In YPD medium, 59A background and 20μM $CuSO_4$ with varying induction times.

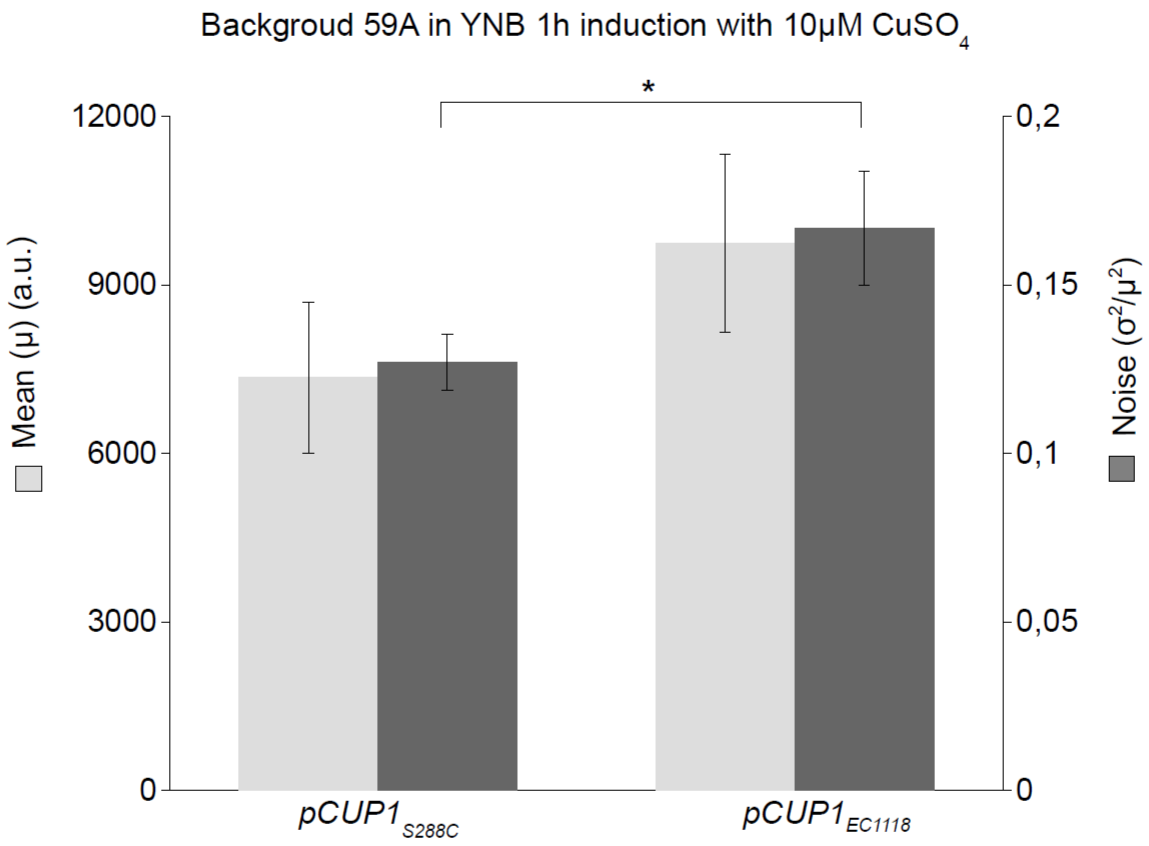

Supplementary Figure 5. The *pCUP1*$_{EC1118}$ promoter variant is noisier than *pCUP1*$_{S288c}$ at the same copper concentration in 59A. Mean and noise values of yEGFP expression conferred by each variant of the *CUP1* promoter were measured in the 59A background and in YNB medium after 1h induction by 10µM $CuSO_4$. A significant statistical difference between mean or noise levels conferred by the promoter variants is represented by (*) when $p<0.05$ in T test.

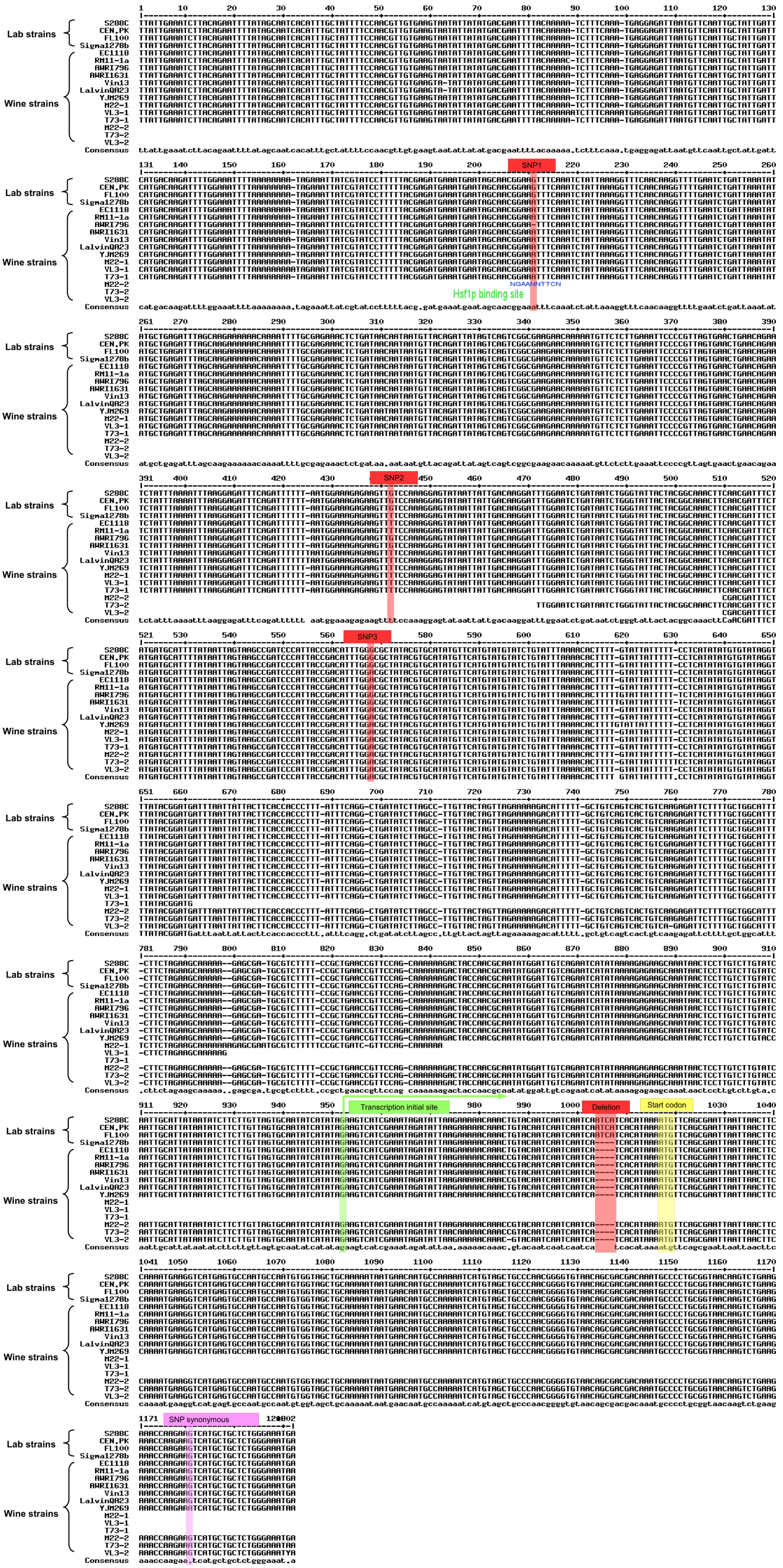
Lab strains
Wine strains
S288C
CEN.PK
FL100
Sigma1278b
EC1118
RM11-1a
AWRI796
AWRI1631
Vin13
LalvinQA23
YJM269
M22-1
VL3-1
T73-1
M22-2
T73-2
VL3-2
Consensus
SNP1
NGAANNTTCN
Hsf1p binding site
SNP2
SNP3
Transcription initial site
Deletion
Start codon
SNP synonymous

Supplementary Figure 6. Alignment of the *CUP1* promoter and ORF sequences of 4 laboratory strains and 10 wine strains. Around 1000 bp upstream the ATG and the ORF are shown. The SNP (numbered from 1 to 3 from upstream to downstream) and deletion between $pCUP1_{S288c}$ and $pCUP1_{EC1118}$ are highlighted in red. The transcription starting site and the start codon are positioned in green and yellow respectively. The Hsf1p binding site discussed in the main text is also highlighted.

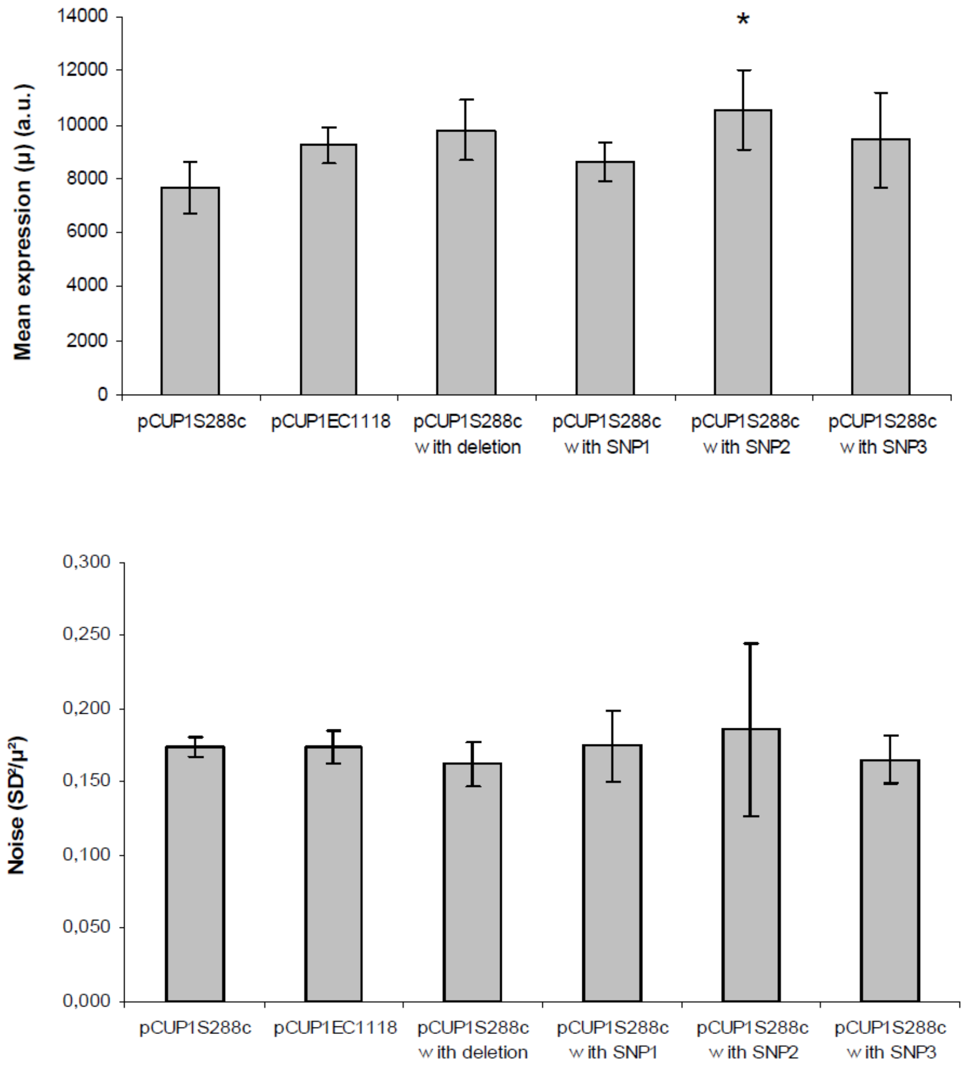

Supplementary Figure 7. Mean and noise levels conferred by the *pCUP1*$_{S288c}$ mutants obtained by directed mutagenesis. Mean expression and noise levels conferred in the 59A strain by the *pCUP1*$_{S288c}$ and *pCUP1*$_{EC1118}$ variants and by the *pCUP1*$_{S288c}$ mutants obtained by directed mutagenesis are shown after induction overnight by 5µM $CuSO_4$. The SNP between *pCUP1*$_{S288c}$ and *pCUP1*$_{EC1118}$ are numbered from 1 to 3 from upstream to downstream. Data are means of three independent cultures, and error bars are standard deviations. A significant statistical difference between mean or noise levels conferred by the promoter variants is represented by (*) when $p<0.05$ in T test.

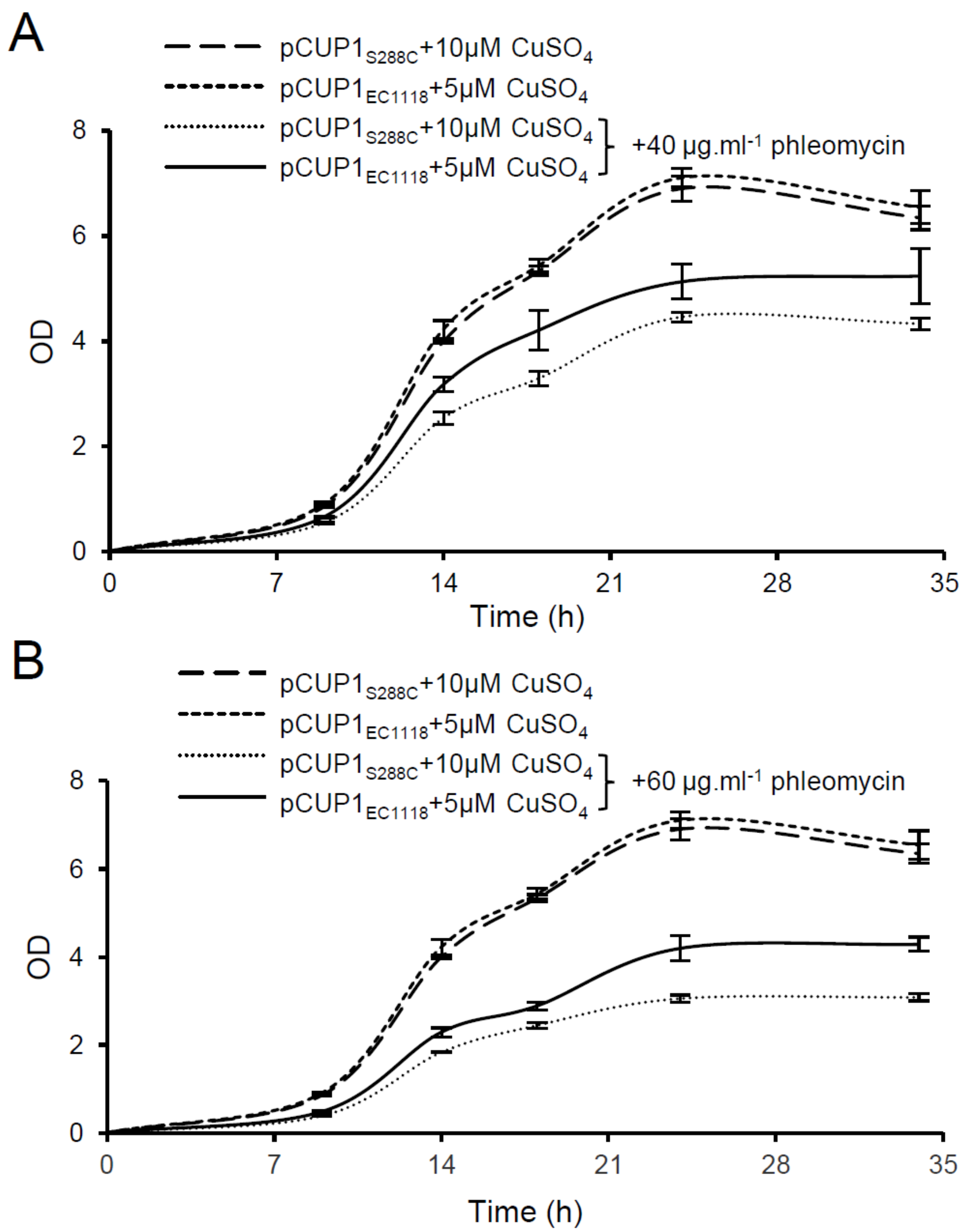

Supplementary Figure 8. The *pCUP1*$_{EC1118}$ and *pCUP1*$_{S288c}$ promoter variants confer distinct selective advantages in various phleomycin concentrations. Strains with *pCUP1*$_{S288c}$ and *pCUP1*$_{EC1118}$ variants fused to *ZeoR* were induced in appropriate $CuSO_4$ concentrations to make the strains express *ZeoR* at similar mean expression levels. Experimental growth time course was performed without phleomycin or at 40 or 60μg/mL phleomycin. Data points are means of three independent cultures, and error bars are standard deviations.

Number	Original clone		1st subclone		2nd subclone	
	Mean	CV	Mean	CV	Mean	CV
1	197,56	131,73	136,77	133,04	157,28	136,89
2	547,87	126,08	477,54	122,03	500,78	128,15
3	220,09	127,08	156,03	125,04	200,24	129,07
4	1076,81	127,59	996,35	125,99	1000,54	128,14
5	163,55	130,91	139,44	127,73	150,25	124,05
6	333,57	125,58	542,11	120,19	500,41	119,41
7	590,38	127,29	541,46	121,41	551,24	122,2
8	776,26	124,24	756	124,21	761,2	121,96
9	1219,14	124,18	1028	124,55	1147,23	129,44
10	737,52	129,9	861,7	128,4	812,47	127,75
11	251,22	124,81	242,59	118,79	224,23	121,79
12	424,14	124,81	404,44	123,59	415,47	120,28
13	238,95	137,21	216,06	140,75	227,13	135,37
14	232,69	130,66	345,12	128,58	300,14	125,05
15	390,62	127,36	254,92	122,69	354,32	127,42
16	230,11	132,05	224,6	131,36	231,21	127,17
17	574,99	128,5	557,57	121,12	521,36	129,83
18	667,87	126,27	606,06	120,9	603,12	122,77
19	855,96	129,06	794,33	126,42	800,47	128,39
20	211,03	127,27	209,08	126,08	221,33	123,75
21	790,47	132,04	1030,76	125,18	853,13	131,19
22	191,43	126,69	197,84	126,1	199,78	121,01
23	947,8	125,76	886,84	123,85	900,85	126,91
24	178,32	127,18	181,52	120,32	178,58	122,77
25	710,52	137,43	705,61	133,33	712,33	135,04
26	584,97	135,66	498,29	135,09	556,32	130,56
27	143,72	129,07	107,27	126,27	133,24	127,36
28	507,99	131,62	603,89	130,74	557,36	137,62
29	978,35	129,81	560,2	124,77	887,12	129,37
30	676,2	130,56	588,01	122,89	624,33	123,18
31	248,13	126,06	243,94	120,08	257,14	123,58

32	34,4	121,22	34,63	123,85	35,47	125,55
33	77,42	117,45	76,48	116,24	75,44	113,46
34	27,88	113,75	27,91	112,51	28,11	110,16
35	30,32	120,76	31,74	128,46	32,21	123,91
36	21,53	122,67	24,4	124,6	23,37	126,55
37	67,01	116,44	65,99	116,94	66,89	114,38
38	28,47	112,87	27,93	114,38	27,97	115,45
39	54,48	118,65	57,42	115,05	58,34	111,73
40	62,3	119,07	67,32	120,76	66,27	125,91
41	37,01	116,95	32,36	119,72	33,24	112,17
42	55,03	122,16	58,31	124,42	53,21	126,31
43	32,42	123,41	28,47	129,5	31,36	120,64
44	47,18	119,94	35,17	120,61	45,24	121,69
45	82,44	114,89	85,63	113,94	84,53	119,52
46	82,25	114,34	84,95	118,8	83,65	114,59
47	46,11	120,2	42,5	115,78	75,37	119,63
48	55,97	114,06	53	104,77	55,38	110,61
49	85,53	122,54	89,8	129,66	88,15	124,9
50	40,36	113,98	38,8	115,57	40,17	117,96
51	32,26	117,49	29,97	112,52	30,25	118,33
52	28,35	115,45	25,68	116,84	27,11	121,48
53	54	118,11	55,88	114,97	53,47	117,2
54	45,84	120,3	38,15	120,08	43,26	127,06
55	67,4	116,82	68,92	115,94	65,17	113,19
56	40,49	121,98	43,84	122,44	44,71	121,81
57	95,31	115,18	90,65	117,53	93,31	113,24
58	79,21	119,4	71,51	116,26	77,84	116,95
59	96,32	120,31	90,21	121,52	93,86	119,44
60	66,28	117,68	66,13	118,93	65,14	113,58
61	28,35	123,4	25,05	122,44	27,03	121,99
62	69,02	114,03	61,55	114,69	68,61	113,5
63	50,01	112,15	52,33	115,68	53,14	115,8
64	66,84	113,08	69,2	115,47	67	115,54

65	55,45	111,27	60,12	118,67	57,15	115,04
66	43,12	123,91	45,28	125,38	47	123,4
67	67,87	112,2	69,64	113,8	68,9	106,1
68	26,95	122,82	23,42	124,38	25,29	122,12
69	44,39	123,09	43,07	115,64	45,99	118,76
70	33,52	124,04	35,37	124,98	36,18	127,17
71	41,58	113,72	44,38	119,81	43,35	115,32
72	41,84	114,55	42,52	112,5	43,17	118,86
73	56,04	119,29	51,22	115,3	55,31	113,98
74	33,31	117,31	31,6	115,46	35,87	116,04
75	63,67	119,6	58,66	118,44	66,47	118,02
76	33,99	116,36	38,07	116,38	35,69	115,98
77	40,36	111,99	42,63	117,35	43,5	110,75
78	32,05	122,05	31,98	123,34	35,36	124
79	30,48	115,3	38,44	117,08	35,22	112,24
80	81,34	118,32	87,92	111,06	85,99	116,27
81	32,42	115,57	35,32	119,16	34,82	113,92
82	81,58	122,46	80,43	128,27	83,26	124,37
83	30,19	119,35	33,39	115,78	31,28	113,32
84	40,55	122,55	35,7	124,29	41,73	121,73
85	43,12	115,73	45,64	119,69	46,17	119,87
86	51,25	119,4	56,65	116,69	55,42	116,09
87	37,58	117,03	30,43	119,23	35,4	118,95
88	41,65	117,89	39,75	117,18	41,26	116,2
89	39,32	114,55	35,37	114,8	37,76	117,81
90	33,52	115,87	36,43	111,56	34,58	114,46
91	50,01	116,71	55,59	113,03	52,47	118,14
92	42,94	120,73	37,04	122,73	41,82	128,47
93	69,02	112,05	61,64	110,6	65,77	115,64
94	55,45	113,89	53,02	115,64	54,78	110,38
95	32,28	121,69	27,62	115,35	30,54	121,26
96	56,73	122,18	51,71	128,45	57,17	124,96
97	33,99	124,14	36,68	121,66	34,32	127,36

Supplementary Table 1. Mean and CV values of 2 sub-clones re-isolated from each sequenced clone. The coefficient of correlation between the values of the initial clones and their sub-clones, and between the sub-clones is calculated.

Clone	Number of cells	Mean	CV	Number of cells-small gate	Mean-small gate	CV-small gate	CV reduction (%)
28-G4-01	99060	110,15	193,33	39475	120,2	128,64	0,335
28-H3-02	98935	224,6	175,75	54986	151,47	108,87	0,381
30-BE11-01	99036	1030,76	150,58	55414	718,37	117,55	0,219
30-BE11-02	99238	1036,96	148,86	56247	739,07	118,23	0,206
						Mean CV reduction:	0,285

Supplementary Table 2. Mean and CV values of some clones showing noisy yEGFP expression before and after sub-gating. The cell number in the initial gate and in the sub-gate on the most homogenous part of the population is also given as well as the CV reduction for each clone.

Supplementary Table 3 (see attached file). Sequencing results on 97 clones showing noisy yEGFP expression with mapping to the S288c genome.

Supplementary Table 4 (see attached file). List of the genetic variations detected in 37 of the 50 sequenced genomic fragments containing known promoters. The reference (Ref) genome is S288c and the alternative genome (Alt) is EC1118.

Transcription Factor	Consensus	Position	Strand
**Target Sequence: pCUP1S288c** (size 1000)			
Ash1p	YTGAT	-878	F
Ash1p	YTGAT	-755	F
Ash1p	YTGAT	-700	F
Ash1p	YTGAT	-526	F
Ash1p	YTGAT	-311	F
Ash1p	YTGAT	-12	R
Ash1p	YTGAT	-16	R
Ash1p	YTGAT	-20	R
Ash1p	YTGAT	-104	R
Azf1p	AAGAAAAA	-728	F
Azf1p	AAGAAAAA	-44	F
Cat8p, Sip4p	YCCNYTNR RCCGN	-199	F
Cup2p	HTHNNGCT GD	-317	F
Cup2p	HTHNNGCT GD	-274	F
Cup2p	HTHNNGCT GD	-245	F
Cup2p	HTHNNGCT GD	-201	F
Cup2p	HTHNNGCT GD	-175	R
Cup2p	GCGTCTTT TCCGCTGA	-207	F
Cup2p	TCTTTTGCT G	-246	F
Cup2p	TCTTTTTTG CTG	-172	R
Fkh1p, Fkh2p	RYMAAYA	-130	F
Fkh1p, Fkh2p	RYMAAYA	-539	R
Fkh1p, Fkh2p	RYMAAYA	-873	R
Gcn4p	TGATTCA	-877	F
Gcn4p	TGACTGA	-258	R
Gcn4p	TGACTMT	-669	R
Gcr1p	CTTCC	-792	R
Gcr1p	CWTCC	-345	R
Gcr1p	CWTCC	-792	R
Hac1p	CCAGC	-185	F
Hac1p	CCAGC	-235	R
Hsf1p	NGAANNTT CN	-797	F
Hsf1p	NGAANNTT CN	-787	R
Hsf1p	NTTCNNGA AN	-230	F

Transcription Factor	Consensus	Position	Strand
**Target Sequence: pCUP1EC1118** (size 996)			
Ash1p	YTGAT	-874	F
Ash1p	YTGAT	-751	F
Ash1p	YTGAT	-696	F
Ash1p	YTGAT	-522	F
Ash1p	YTGAT	-307	F
Ash1p	YTGAT	-12	R
Ash1p	YTGAT	-16	R
Ash1p	YTGAT	-100	R
Azf1p	AAGAAAAA	-724	F
Azf1p	AAGAAAAA	-40	F
Cat8p, Sip4p	YCCNYTNR RCCGN	-195	F
Cup2p	HTHNNGCT GD	-313	F
Cup2p	HTHNNGCT GD	-270	F
Cup2p	HTHNNGCT GD	-241	F
Cup2p	HTHNNGCT GD	-197	F
Cup2p	HTHNNGCT GD	-171	R
Cup2p	GCGTCTTT TCCGCTGA	-203	F
Cup2p	TCTTTTGCT G	-242	F
Cup2p	TCTTTTTTG CTG	-168	R
Fkh1p, Fkh2p	RYMAAYA	-126	F
Fkh1p, Fkh2p	RYMAAYA	-535	R
Fkh1p, Fkh2p	RYMAAYA	-869	R
Gcn4p	TGATTCA	-873	F
Gcn4p	TGACTGA	-254	R
Gcn4p	TGACTMT	-665	R
Gcr1p	CWTCC	-341	R
Hac1p	CCAGC	-181	F
Hac1p	CCAGC	-231	R
Hsf1p	NGAANNTT CN	-793	F
Hsf1p	NGAANNTT CN	-783	R
Hsf1p	NTTCNNGA AN	-226	F
Hsf1p	NTTCNNGA AN	-216	R
Mcm1p	DCCYWWW NNRG	-769	R
Mcm1p	CCYWWWN NRG	-770	R

Supplementary Table 5. Transcription factors binding sites present in the *pCUP1*$_{S288c}$ and *pCUP1*$_{EC1118}$ variants according to Yeastract. (http://www.yeastract.com/). Their position is given from the start codon.

Nom	Genotype
JF1581	BY4720 *MATα lys2Δ0 trp1Δ63 ura3Δ0*
JF2350	59A *MATα Δamn1-loxP*
JF1094	CEN.PK *MATa ura3-52*
JA0111	JF1581 *leu2::pBMH1*$_{S288c}$*-GFP*
JA0112	JF1581 *leu2::pBMH1*$_{EC1118}$*-GFP*
JA0113	JF1581 *leu2::pBMH2*$_{S288c}$*-GFP*
JA0114	JF1581 *leu2::pBMH2*$_{EC1118}$*-GFP*
JA0115	JF1581 *leu2::pCAN1*$_{S288c}$*-GFP*
JA0116	JF1581 *leu2::pCAN1*$_{EC1118}$*-GFP*
JA0117	JF1581 *leu2::pCUP1*$_{S288c}$*-GFP*
JA0118	JF1581 *leu2::pCUP1*$_{EC1118}$*-GFP*
JA0119	JF1581 *leu2::pHAC*$_{S288c}$*-GFP*
JA0120	JF1581 *leu2::pHAC1*$_{EC1118}$*-GFP*
JA0121	JF1581 *leu2::pGNP1*$_{S288c}$*-GFP*
JA0122	JF1581 *leu2::pGNP1*$_{EC1118}$*-GFP*
JA0123	JF1581 *leu2::pYCK2*$_{S288c}$*-GFP*
JA0124	JF1581 *leu2::pYCK2*$_{EC1118}$*-GFP*
JA0125	JF1581 *leu2::pAGP2*$_{S288c}$*-GFP*
JA0126	JF1581 *leu2::pAGP1*$_{EC1118}$*-GFP*
JA0511	JF2350 *leu2::pBMH1*$_{S288c}$*-GFP*
JA0512	JF2350 *leu2::pBMH1*$_{EC1118}$*-GFP*
JA0513	JF2350 *leu2::pBMH2*$_{S288c}$*-GFP*
JA0514	JF2350 *leu2::pBMH2*$_{EC1118}$*-GFP*
JA0515	JF2350 *leu2::pCAN1*$_{S288c}$*-GFP*
JA0516	JF2350 *leu2::pCAN1*$_{EC1118}$*-GFP*
JA0517	JF2350 *leu2::pCUP1*$_{S288c}$*-GFP*
JA0518	JF2350 *leu2::pCUP1*$_{EC1118}$*-GFP*
JA0519	JF2350 *leu2::pHAC1*$_{S288c}$*-GFP*
JA0520	JF2350 *leu2::pHAC1*$_{EC1118}$*-GFP*
JA0521	JF2350 *leu2::pGNP1*$_{S288c}$*-GFP*
JA0522	JF2350 *leu2::pGNP1*$_{EC1118}$*-GFP*
JA0523	JF2350 *leu2::pYCK2*$_{S288c}$*-GFP*
JA0524	JF2350 *leu2::pYCK2*$_{EC1118}$*-GFP*
JA0525	JF2350 *leu2::pAGP2*$_{S288c}$*-GFP*
JA0526	JF2350 *leu2::pAGP2*$_{EC1118}$*-GFP*
JA0538	JF2350 *leu2::pCUP1*$_{S288c}$*-Deletion-GFP*
JA0539	JF2350 *leu2::pCUP1*$_{S288c}$*-SNP1-GFP*
JA0540	JF2350 *leu2::pCUP1*$_{S288c}$*-SNP2-GFP*
JA0541	JF2350 *leu2::pCUP1*$_{S288c}$*-SNP3-GFP*
JA0542	JF2350 *leu2::pCUP1*$_{S288c}$*-zeoR-kan*
JA0543	JF2350 *leu2::pCUP1*$_{EC1118}$*-zeoR-kan*

Supplementary Table 6. List of the strains used in the study.

Oligo name	Sequence
pUG35promlessf	TGACCATGATTACGCCAAGCGCGCAATTAACCCTCACTAAAGGGAACAAAAGCTGCGTACGCTGCAGGTCGAC
pUG35promlessr+0	CTAATTCAACCAAAATTGGGACAACACCAGTGAATAATTCTTCACCTTTAGACATTACGTAATCGATGAATTCGAGCTCG
pUG35promlessr+1	TAATTCAACCAAAATTGGGACAACACCAGTGAATAATTCTTCACCTTTAGACATTTACGTAATCGATGAATTCGAGCTCG
pUG35promlessr+2	AATTCAACCAAAATTGGGACAACACCAGTGAATAATTCTTCACCTTTAGACATTTTACGTAATCGATGAATTCGAGCTCG
SeqGenomicFrag	CTTCACCGGAGACAGAAAAT
BMH1-SalI-For	ATTTGTCGACATTTCTATGCAACAAGAATA
BMH1-EcoRI-Rev	CCCGGAATTCTTTTATCTTTAGTTTATCTTTAAC
BMH2-SalI-For(infu)	GCATCTCGAGGTCGAAATAGGGAATCGGTATTTCTG
BMH2-EcoRI-Rev(infu)	CTTTAGACATGAATTTTTTTTTGTTGTAACGGGTAC
CUP1-2-SalI-For	ACGGTCGACACAGAATTTTATAGCAATCAC
CUP1-2-EcoRI-Rev	TTCGGAATTCTTTATGTGATGATTGATTGA
CAN1-SalI-For	ATTGTCGACTGTGTGTATGGGCACAAACC
CAN1-EcoRI-Rev	CCGGAATTCTGCTATGCCTTTTTTTTTTTTTG
YCK2-SalI-For	CATTGTCGACTCTCTCGTACTATTAACATACC
YCK2-EcoRI-Rev	CTCGGAATTCTTTTGGAAAACTATTTTCTT
HAC1-SalI-For	ATCTGTCGACCGTGTTCCACTGTGGAGAGC
HAC1-MefI-Rev	CCGCAATTGAGTGGCGGTTGTTGTCGTAG
GNP1-SalI-For	ATCTGTCGACCGTGTATTTATTTGTAAAACT
GNP1-EcoRI-Rev	ACGGAATTCAATGTGCAATATTTGATATT
GFP-EcoRI-for	GCCCCGGAATTCATGTCTAAAGGTGAAGAATTA
GFP-NotI-Rev	ATTGCGGCCGCTTATTTGTACAATTCATCCA
GFP-EcoRI-for	GCCCCGGAATTCATGTCTAAAGGTGAAGAATTA
GFP-NotI-Rev	ATTGCGGCCGCTTATTTGTACAATTCATCCA
Mut-del-For	CTGTACAATCAATCAATCATCACATAAAATGTTCAGCG
Mut-del-Rev	CGCTGAACATTTTATGTGATGATTGATTGATTGTACAG
Mut-793-For	GATGAAATGAATAGCAACGGAAATTTCAAATCTATTAAAGGTTTC
Mut-793-Rev	GAAACCTTTAATAGATTTGAAATTTCCGTTGCTATTCATTTCATC
Mut-563-For	GATTTTTTAATGGAAAGAGAAGTTTTCCAAAGGAGTATAATTATTGAC
Mut-563-Rev	GTCAATAATTATACTCCTTTGGAAAACTTCTCTTTCCATTAAAAAATC
Mut-437-For	CCCATTACCGACATTTGGACGCTATACGTGCATATGT
Mut-437-Rev	ACATATGCACGTATAGCGTCCAAATGTCGGTAATGGG
ZeoR-EcoR1-For	GGGGGCGGAATTCATGGCCAAGTTGACCAGTGC
ZeoR-Not1-rev	TAAATAAGCGGCCGCTCAGTCCTGCTCCTCGGCCACG
Mut-del-For-New (phosphalated)	TCACATAAAATGTTCAGCGAA
Mut-del-Rev-New (phosphalated)	TGATTGATTGATTGTACAGTTTG
CHECK-PJL2-for	ACATACATAAACATACGCGC
CHECK-PJL2-Rev	TTATCACGTTGAGCCATTAG

Supplementary Table 7. List of the primers used in the study.
